# Depsipeptide Intermediates Interrogate Proposed Biosynthesis of Cereulide, the Emetic Toxin of *Bacillus cereus*

**DOI:** 10.1038/srep10637

**Published:** 2015-05-27

**Authors:** Sandra Marxen, Timo D. Stark, Andrea Rütschle, Genia Lücking, Elrike Frenzel, Siegfried Scherer, Monika Ehling-Schulz, Thomas Hofmann

**Affiliations:** 1 Chair of Food Chemistry and Molecular Sensory Science, Technische Universität München, Lise-Meitner-Straße 34, 85354 Freising, Germany; 2 Department of Microbiology, Central Institute for Food and Nutrition Research, Technische Universität München, 85350 Freising; 3 Functional Microbiology, Institute of Microbiology Department of Pathobiology, University of Veterinary Medicine Vienna, 1210 Vienna, Austria; 4 Chair of Microbial Ecology, Department of Biosciences, WZW, Technische Universität München, 85350 Freising, Germany; 5 Bavarian Center for Biomolecular Mass Spectrometry, Technische Universität München, Gregor-Mendel Strasse 4, 85354 Freising, Germany

## Abstract

Cereulide and isocereulides A-G are biosynthesized as emetic toxins by *Bacillus cereus* via a non-ribosomal peptide synthetase (NRPS) called Ces. Although a thiotemplate mechanisms involving cyclo-trimerization of ready-made D-*O*-Leu-D-Ala-L-*O*-Val-L-Val via a thioesterase (TE) domain is proposed for cereulide biosynthesis, the exact mechanism is far from being understood. UPLC-TOF MS analysis of *B. cereus* strains in combination with ^13^C-labeling experiments now revealed tetra-, octa-, and dodecapeptides of a different sequence, namely (L-*O*-Val-L-Val-D-*O*-Leu-D-Ala)_1-3_, as intermediates of cereulide biosynthesis. Surprisingly, also di-, hexa-, and decadepsipeptides were identified which, together with the structures of the previously reported isocereulides E, F, and G, do not correlate to the currently proposed mechanism for cereulide biosynthesis and violate the canonical NRPS biosynthetic logic. UPLC-TOF MS metabolite analysis and bioinformatic gene cluster analysis highlighted dipeptides rather than single amino or hydroxy acids as the basic modules in tetradepsipeptide assembly and proposed the CesA C-terminal C* domain and the CesB C-terminal TE domain to function as a cooperative esterification and depsipeptide elongation center repeatedly recruiting the action of the C* domain to oligomerize tetradepsipeptides prior to the release of cereulide from the TE domain by macrocyclization.

The endospore-forming bacterium *Bacillus cereus* is a food-borne human pathogen causing diarrhea due to the activity of haemolytic and nonhaemolytic enterotoxins as well as food intoxications induced by the heat-stable, emesis provoking cereulide^1^,^2^. Cereulide (**1**) is a dodecadepsipeptide composed of six α-amino acid and six α-hydroxy acid moieties arranged in three repeating tetradepsipeptide units, namely [D-*O*-Leu-D-Ala-L-*O*-Val-L-Val]_3_ (Fig. 1)^3^,^4^.

Biosynthesis of fungal or bacterial cyclooligomer depsipeptides, such as the cyclohexadepsipeptide enniatin from *Fusarium* species or the cyclooctadepsipeptide bassianolide from *Beauveria bassiana*, as well as the cyclodecadepsipeptide valinomycin from *Streptomyces* species or the cyclododecadepsipeptide cereulide from *B. cereus* is performed by large multi-domain non-ribosomal peptide synthetases (NRPS)^5^,^6^,^7^,^8^. In *B. cereus*, the cereulide non-ribosomal peptide synthetase (Ces NRPS) is encoded by the polycistronically transcribed *ces* gene cluster, which is located on a mega plasmid that is sharing its backbone with the *B. anthracis* toxin plasmid pX01^9^,^10^,^11^. Besides the structural genes for the synthetase (*cesA* and *cesB*), the ces gene locus comprises a putative hydrolase (*cesH*), a phosphopanthetheinyl transferase (*cesP*), a type II thioesterase (*cesT*), as well as a putative ABC transporter (*cesC/D*)^9^.

The biosynthesis of cereulide is proposed to follow the general principle of NRPS multi-enzyme complexes that show a modular organization. Every single module, delivering a specific monomer to the growing peptide, can be subdivided into conserved domains that carry all essential information for recognition, activation, and modification of the substrate. The adenylation (A) domain is responsible for specific recognition and activation of the precursors by adenylation using ATP as co-substrate. A peptidyl carrier protein (PCP) captures the precursor adenylates as covalent thioesters via a phosphopantetheine linker^12^. A characteristic feature of CesA and CesB is the existence of two A domains which recognize, select, and reduce α-ketocarboxylic acids to α-hydroxyacyl-*S*-pantetheinyl intermediates. The chiral reduction step is catalyzed by ketoreductase (KR) domains embedded in the corresponding A domains CesA1 and CesB1, respectively. Chain elongation of the intermediate is mediated by a condensation (C) domain catalyzing the nucleophilic attack of the downstream PCP-bound acceptor monomer on the activated thioester of the upstream PCP-bound donor monomer^13^, thus giving rise to the dipeptide intermediate D-*O*-Leu-D-Ala-*S*-PCP. Due to their enantioselectivity, they are also thought to act as selective filters, facilitating the production of NRPS products in a directed manner^14^. The CesA’s terminal C domain is proposed to work in *trans* with the D-*O*-Leu-D-Ala peptide as donor and the hydroxy group of the CesB1 PCP-thioester bound L-*O*-Val, followed by CesB2 PCP-bound L-Val as nucleophilic acceptors^8^. Elongation to the D-*O*-Leu-D-Ala-L-*O*-Val-L-Val chain is presumably followed by transfer to the hydroxy group of a serine of the thioesterase (TE) domain, while a second tetradepsipeptide builds up on the adjacent PCP of CesB. The thioesterase (TE) domain is able to release the target peptide by either hydrolysis or cyclization^12^,^15^,^16^ and is supposed to condense tetradepsipeptides, first to produce an octadepsipeptidyl-*O*-TE and then a decadepsipeptidyl-*O*-TE, followed by macrolactonization to form the cyclic twelve residue product cereulide^8^,^9^.

Interestingly, recent UPLC-TOF MS profiling of 78 *B. cereus* strains showed a high variability in cereulide production and indicated a unique pattern of 18 previously unknown cereulide variants, among which isocereulide A-G (**2**-**8**; Fig. 1) could be unequivocally identified by means of ion-trap MS^n^ sequencing, ^13^C-labeling experiments, and post-hydrolytic dipeptide and enantioselective amino acid analysis^17^,^18^. The structures of some isocereulides differed from that of cereulide by substitution of L-*O*-Val by L-*O*-Leu (isocereulide A), L-Val by L-Ala (isocereulide D), D-Ala by D-Ser (isocereulide C), and D-*O*-Leu by D-*O*-Val (isocereulide B), thus indicating a relaxed substrate specificity of the A domains and an inefficient proof reading by CesT^18^. However, the structures of isocereulides E (**6**) and F (**7**), as well as isocereulide G (**8**), which shows another constitutional arrangement of the depsidipeptides D-*O*-Leu-D-Ala and L-*O*-Val-L-Val when compared to the isobaric cereulide, violate the canonical NRPS biosynthesis logic as suggested for cereulide production.

To gain novel insights into the non-ribosomal peptide assembly leading to cereulide and isocereulides, the presence of depsipeptide intermediates in a variety of *B. cereus* strains was screened by UPLC-TOF MS and their abundance was correlated with the cereulide productivity of different *B. cereus* strains. In addition, the *ces* gene locus was reinvestigated using bioinformatic approaches.

## Results and discussion

### UPLC-TOF MS metabolite analysis of *B. cereus* strains

**Figure 2:**
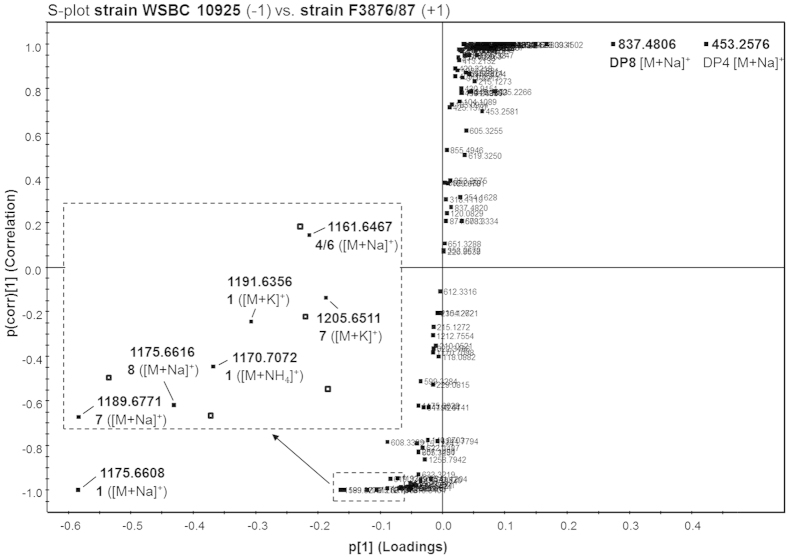
S-Plot of UPLC-TOF MS data of cereulide high producing strain WSBC 10925 vs. low-medium producer F3876/87 with metabolite assignment for cereulide (**1**) with *m/z* 1175.6608 ([M+Na]^+^), *m/z* 1191.6356 ([M+K]^+^) and *m/z* 1170.7072 ([M+NH_4_]^+^), isocereulide F (**7**) with *m/z* 1189.6771 ([M+Na]^+^) and *m/z* 1205.6511 ([M+K]^+^), isocereulide G (**8**) with *m/z* 1175.6616 ([M+Na]^+^), isocereulide B and E (**4**, **6**) with *m/z* 1161.6467 ([M+Na]^+^), and the open-chain tetradepsipeptide (**DP4**; *m/z* 453.2576; C_21_H_38_N_2_O_7_Na, -1.5 ppm) and octadepsipeptide (**DP8**; *m/z* 837.4806; C_40_H_70_N_4_O_13_Na, -4.7 ppm).

In order to investigate differences in the metabolite profile of cereulide low-medium and high producers, ethanol extracts were prepared from cultures of strains WSBC 10925 and F3876/87, respectively, and analyzed by means of UPLC-ESI TOF MS. To visualize similarities and differences between the samples, S-plots of data pairs of accurate mass and retention time of each metabolite were calculated based on orthogonal partial least squares discriminant analysis (OPLS-DA). As the y-axis of the S-plot denotes confidence of a metabolite’s contribution to the group difference and the x-axis denotes the contribution of a particular metabolite to the group difference, the S-plot indicated the ion *m/z* 1175.6608 to show by far the highest difference in abundance in high producer strain WSBC 10925, whereas the ions *m/z* 453.2576 and *m/z* 837.4806 showed significantly higher abundance in the cereulide low-medium producer strain F3876/87 (Fig. 2). The pseudo molecular ion *m/z* 1175.6608 ([M+Na]^+^) was unequivocally identified as sodium adduct (C_57_H_96_N_6_O_18_Na) of cereulide (**1**) and, together with the corresponding [M+K]^+^ (*m/z* 1191.6356) and [M+NH_4_]^+^ ion (*m/z* 1170.7072), was confirmed by co-chromatography with a purified cereulide reference^17^,^18^. Moreover, the pseudomolecular ions *m/z* 1189.6771 ([M+Na]^+^) and *m/z* 1205.6511 ([M+K]^+^) were assigned as isocereulide F (**7**), *m/z* 1175.6616 ([M+Na]^+^) as isocereulide G (**8**), and *m/z* 1161.6467 ([M+Na]^+^) as a mixture of the isobaric isocereulides B (**4**) and E (**6**) by means of co-chromatography with purified reference materials.

On the basis of accurate mass data, the sum formula of the ions *m/z* 453.2576 and 837.4806, detected with high abundance in strain F3876/87, were calculated to be C_21_H_38_N_2_O_7_Na (–1.5 ppm) and C_40_H_70_N_4_O_13_Na (–4.7 ppm), respectively. Interestingly, these sum formula were in good agreement with the ethyl esters of the open-chain tetradepsipeptide D-*O*-Leu-D-Ala-L-*O*-Val-L-Val and the octadepsipeptide (D-*O*-Leu-D-Ala-L-*O*-Val-L-Val)_2_ proposed to be key intermediates in cereulide biosynthesis (Fig. 2)^8^.

### Structure determination of candidate depsipeptide esters

**Figure 3:**
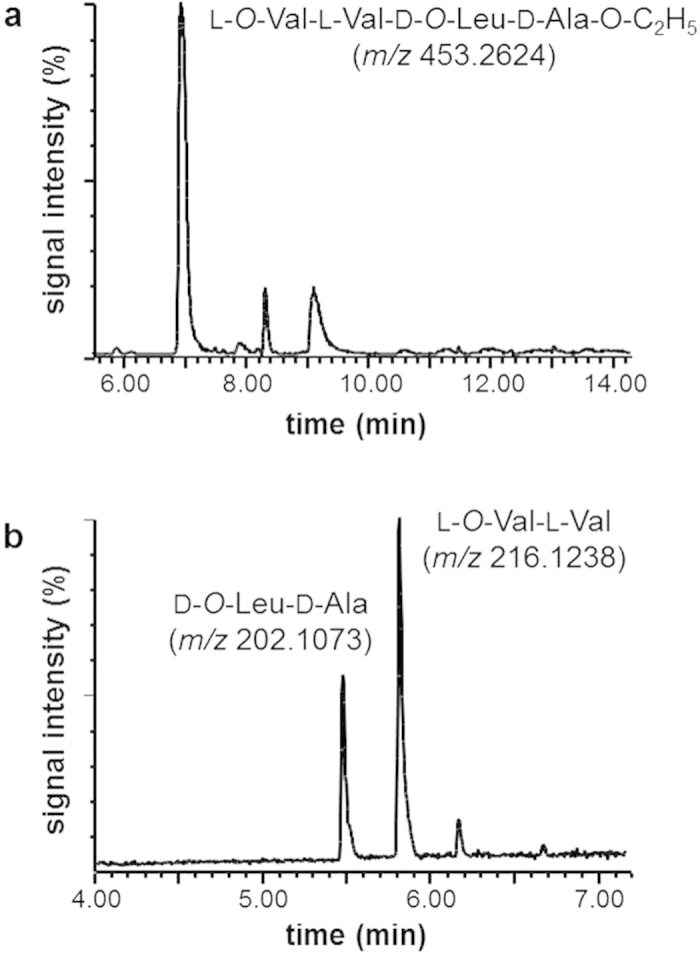
UPLC-TOF MS analysis of (**a**) isolated tetradepsipeptide (**DP4**; *m/z* 453.2576; ESI^+^) and (**b**) dipeptides D-*O*-Leu-D-Ala (*m/z* 202.1073, [M-H]^–^) and L-*O*-Val-L-Val (*m/z* 216.1238, [M-H]^–^) after alkaline hydrolysis.

In order to determine the chemical structures of the candidate depsipeptide esters detected, the fraction containing the target peptide esters with *m/z* 453.2576 and 837.4806, respectively, were isolated from the ethanolic extract of strain F4810/72 by means of RP-HPLC. As an example the target compound with *m/z* 453.2576 is shown in Fig. 3a. Alkaline hydrolysis of the isolated depsipeptides produced dipeptides as reported recently for cereulide and isocereulides^18^. UPLC-TOF MS analysis of the alkaline hydrolysate of the candidate tetradepsipeptide ethyl ester (Fig. 3b) and comparison with synthetic reference peptides led to the identification of D-*O*-Leu-D-Ala (*m/z* 202.1073, [M-H]^–^) and L-*O*-Val-L-Val (*m/z* 216.1238, [M-H]^–^), thus matching the cleavage peptides of cereulide’s D-*O*-Leu-D-Ala-L-*O*-Val-L-Val tetradepsipeptide motif^18^. Similarly, alkaline hydrolysis of the candidate octadepsipeptide (*m/z* 837.4806), followed by UPLC-TOF MS analysis, also revealed D-*O*-Leu-D-Ala (*m/z* 202.1073, [M-H]^–^) and L-*O*-Val-L-Val (*m/z* 216.1238, [M-H]^–^) as cleavage peptides as expected to be released from (D-*O*-Leu-D-Ala-L-*O*-Val-L-Val)_2_-OEt (data not shown).

**Figure 4:**
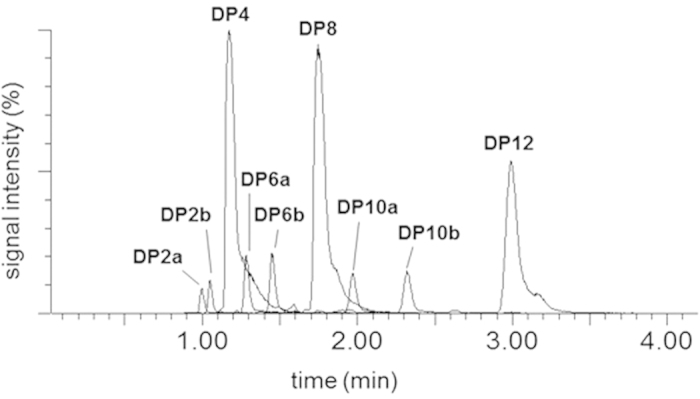
UPLC-TOF MS (ESI^+^) analysis of a MeOH extract of *B. cereus* strain F3876/87 with peak assignment of two didepsipeptide methyl esters with *m/z* 240.1212 (**DP2a**; C_10_H_19_NO_4_Na, +0.1 ppm) and *m/z* 254.1368 (**DP2b**; C_11_H_21_NO_4_Na, +2.0 ppm), the tetradepsipeptide methyl ester with *m/z* 453.2576 (**DP4**; C_21_H_38_N_2_O_7_Na, -1.5 ppm), two hexadepsipeptide methyl esters with *m/z* 624.3472 (**DP6a**; C_29_H_51_N_3_O_10_Na, +0.8 ppm) and *m/z* 638.3629 (**DP6b**; C_30_H_53_N_3_O_10_Na, +0.9 ppm), the octadepsipeptide methyl ester (**DP8**; *m/z* 837.4806; C_40_H_70_N_4_O_13_Na, -4.7 ppm), two decadepsipeptide methyl esters with *m/z* 1008.5733 (**DP10a**; C_48_H_83_N_5_O_16_Na, +0.7 ppm) and *m/z* 1022.5889 (**DP10b**; C_49_H_85_N_5_O_16_Na, +0.8 ppm), and the dodecadepsipeptide methyl ester (**DP12**) with *m/z* 1207.6941 (C_58_H_100_N_6_O_19_Na, +1.0 ppm).

To unequivocally confirm the proposed structure of the ethyl ester of the tetradepsipeptide (*m/z* 453.2576) and the octadepsipeptide (*m/z* 837.4806), respectively, in a first set of experiments cell pellets of strain F3876/87 were extracted with MeOH or MeCN/H_2_O (70/30, v/v) instead of EtOH and the extract was analyzed for depsipeptide methyl esters as well as the free depsipeptides by UPLC-TOF MS. UPLC-TOF MS analysis of the MeOH extract clearly revealed the methyl esters of the expected tetra- (**DP4**) and octadepsipeptide (**DP8**) and, in addition, the methyl ester of the dodecadepsipeptide (**DP12**) with *m/z* 1207.6941 (C_58_H_100_N_6_O_19_Na, +1.0 ppm) as the predominant compounds (Fig. 4). Sursprisingly, also two didepsipeptides with *m/z* 240.1212 (**DP2a**; C_10_H_19_NO_4_Na, +0.1 ppm) and *m/z* 254.1368 (**DP2b**; C_11_H_21_NO_4_Na, +2.0 ppm), two hexadepsipeptides with *m/z* 624.3472 (**DP6a**; C_29_H_51_N_3_O_10_Na, +0.8 ppm) and *m/z* 638.3629 (**DP6b**; C_30_H_53_N_3_O_10_Na, +0.9 ppm), and two decadepsipeptide methyl esters with *m/z* 1008.5733 (**DP10a**; C_48_H_83_N_5_O_16_Na, +0.7 ppm) and *m/z* 1022.5889 (**DP10b**; C_49_H_85_N_5_O_16_Na, +0.8 ppm) were identified at somewhat lower abundance (Fig. 4, Table 1). Also, extraction of the cell pellets with MeCN/H_2_O (70/30, v/v) released the corresponding free oligodepsipeptides **DP2** to **DP12** (Table 1).

**Figure 5:**
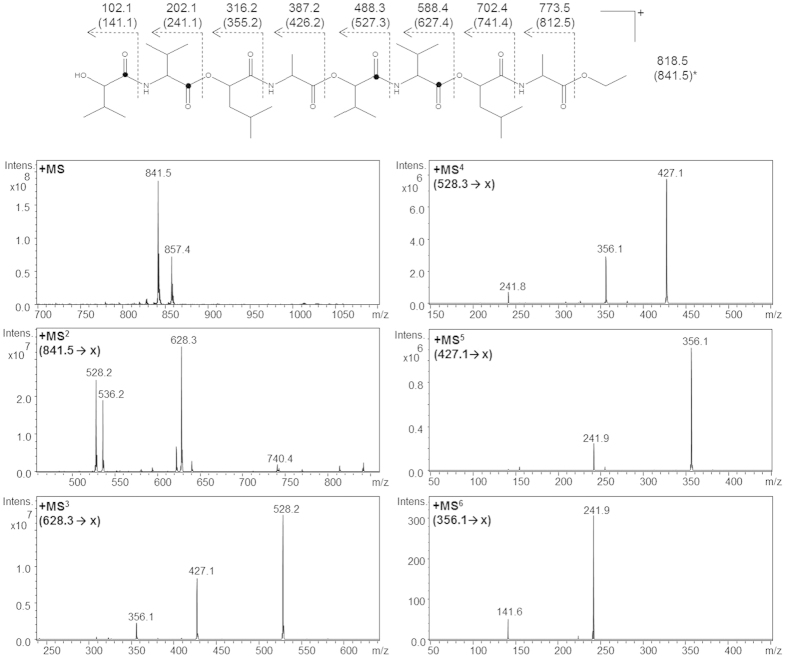
MS^n^ sequencing of the ^13^C_4_-octadepsipeptide ethyl ester (upper masses: calculated, lower masses given in parenthesis: measured [M+K]^+^ ions); •: ^13^C-labeling of α-hydroxy isovaleric acid and valine.

These findings were further confirmed by a ^13^C-labeling experiment. Cultivation of strain F4810/72 in MOD-medium supplemented with ^13^C_1_-valine revealed ^13^C_6_-labeled cereulide^17^,^18^,^19^ as well as the ethyl esters of the ^13^C_2_-labeled tetradepsipeptide (**DP4**), a ^13^C_2_- and a ^13^C_4_-labeled hexadepsipeptide (**DP6a/b**), a ^13^C_4_-labeled octadepsipeptide (**DP8**), a ^13^C_4_- and a ^13^C_6_-labeled decadepsipeptide (**DP10a/b**), as well as a ^13^C_6_-labeled dodecadepsipeptide (**DP12**) released from the cell pellet upon ethanol extraction (Table 1). To confirm the constitutional arrangement of the amino acids and hydroxycarboxylic acids in the target depsipeptides, sequence analyses were performed by MS^n^-experiments. Using the ^13^C_4_-octadepsipeptide ethyl ester as an example, MS fragmentation of the pseudomolecular ion (*m/z* 841.5, [M+Na]^+^) revealed specific fragment ions for the hexadepsipeptide L-*O*-Val-L-Val-D-*O*-Leu-D-Ala-L-*O*-Val-L-Val (*m/z* 841.5 [M+Na]^+^ → 628.3, 528.2 [M+K]^+^) released after cleavage of D-*O*-Leu-D-Ala (Fig. 5). After isolation of the ion *m/z* 628.3, the recorded MS^3^ spectrum showed specific fragment ions indicating the cleavage of the amino acid valine (*m/z* 628.3 [M+K]^+^ → 528.2, [M+K]^+^) or the dipeptide L-*O*-Val-L-Val (*m/z* 628.3 [M+K]^+^ → 427.1 [M+K]^+^) to release the tetradepsipeptide L-*O*-Val-L-Val-D-*O*-Leu-D-Ala which could also be detected in the MS^4^ spectrum after isolation of the fragment ion *m/z* 528.2. Further fragmentation of the ion *m/z* 427.1 ([M+K]^+^ → *m/z* 356.1 → *m/z* 241.9; MS^5^ spectrum) and m/z 356.1 (M+K]^+^ → *m/z* 241.9 → *m/z* 141.6; MS^6^ spectrum), respectively, revealed a L-*O*-Val-L-Val-D-*O*-Leu-D-Ala sequence of the tetradepsipeptide and unequivocally confirmed two ^13^C-labeled carbon atoms in α-hydroxy isovaleric acid (L-*O*-Val) and valine (L-Val), respectively (Fig. 5). Similar MS^n^ experiments enabled the sequence elucidation of the other peptides and peptide esters (Table 1, and Supplementary Information). Most intriguingly, the amino acid sequences found for the open-chain tetra- and octadepsipeptides were L-*O*-Val-L-Val-D-*O*-Leu-D-Ala and (L-*O*-Val-L-Val-D-*O*-Leu-D-Ala)_2_ and did not match with D-*O*-Leu-D-Ala-L-*O*-Val-L-Val and (D-*O*-Leu-D-Ala-L-*O*-Val-L-Val)_2_ proposed as intermediary depsipeptides in cereulide biosynthesis^8^.

As the identified di-, hexa-, and decadepsipeptides are not in line with the proposed biosynthetic assembly of three ready-made tetradepsipeptides^8^,^9^, these findings prompted us to ask whether the candidate depsipeptide esters are released from chemically unstable NRPS-bound PCP-*S*-ester or TE-*O*-ester intermediates of cereulide biosynthesis upon transesterification with ethanol, or are formed as artifacts by esterification of free depsipeptides or as cereulide degradation products released upon ethanolysis. In a first set of experiments, the *B. cereus* cell pellet was exhaustively extracted with MeCN to separate cereulide, followed by an extraction of the cereulide-free residue with MeOH instead of EtOH. UPLC-TOF MS analysis confirmed cereulide as the major compound in the MeCN extract, whereas the ions *m/z* 439.2420, 823.4681 and 1207.6941, corresponding to candidate peptide methyl esters, were detected in the MeOH extract (Table 1). UPLC-TOF MS analysis of methanolic or ethanolic solutions containing the synthesized dipeptides D-*O*-Leu-D-Ala and L-*O*-Val-L-Val, or purified cereulide did not reveal even trace amounts of the candidate peptide methyl or ethyl esters after incubation for 4 days at room temperature (data not shown). These data show the low reactivity of the free depsipeptides and confirm the chemical stability of cereulide as reported earlier^20^,^21^,^22^.

In a second set of experiments, a ^13^C-labeling study was performed to confirm that the open-chain depsipeptides are not formed from ready-made cereulide upon enzymatic cleavage. A *Bacillus cereus* culture was incubated in the presence of ^13^C_6_-cereulide for up to 40 h and ^13^C_6_-cereulide as well as non-labeled cereulide were analyzed by means of UPLC-TOF-MS. The data clearly showed that cereulide is produced *de-novo* as expected while the spiked ^13^C_6_-labeled cereulide did not decline significantly over time, thus demonstrating that cereulide is not enzymatically degraded by *B. cereus*. This is well in line with the observation that none of the ^13^C-labelled tetra-, octa- and depsipeptide ethyl esters were detectable in the ^13^C_6_-cereulide-spiked incubations by means of LC-TOF MS. These findings exclude the open-chain depsipeptides as cereulide degradation products and further strengthen the proposed release of the target depsipeptide esters by transesterification of biosynthetic intermediates like NRPS-bound PCP-*S*-esters and TE-*O*-esters, respectively.

### Quantitative *B. cereus* strain screening for cereulide productivity and NRPS-bound depsipeptide intermediates

**Figure 6:**
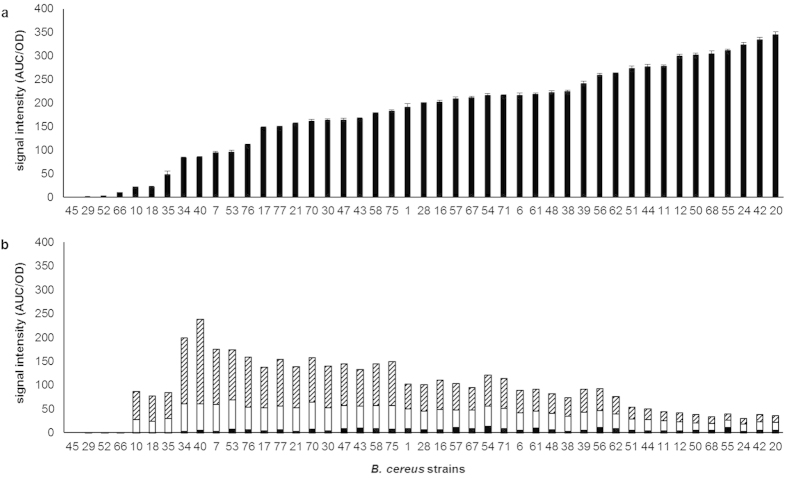
UPLC-ESI-TOF MS analysis of (**a**) cereulide and (**b**) the ethyl esters of tetradepsipeptide (dashed bar), octadepsipeptide (white bar) and dodecadepsipeptide (black bar) in 42 *B. cereus* strains.

A selection of 42 *B. cereus* strains adjusted in optical density (OD) were analyzed by UPLC-TOF MS for cereulide and the major tetra- (**DP4**), octa- (**DP8**), and dodecadepsipeptide ethyl esters (**DP12**) released upon ethanol extraction of the cell pellet^17^. No/low producing strains (no. 45, 29, 52, 66) neither produced elevated levels of cereulide, nor could depsipeptide intermediates be detected in significant amounts (Fig. 6). Intriguingly, cereulide productivity in medium to high producers seem to be inversely correlated with the total abundance of released tetra-, octa-, and dodecadepsipeptide esters measured as a proxy for NRPS-bound depsipeptides. Moreover, with decreasing cereulide productivity higher amounts of tetradepsipeptide esters were liberated when compared to the octa- and dodecadepsipeptide esters (Fig. 6). These data are first evidence that cereulide high producers rapidly oligomerize the precursor depsipeptides followed by subsequent cyclization, whereas less productive strains seem to accumulate NRPS-bound intermediates at the stage of tetradepsipeptides, only slowly converting them to higher oligomers.

### **G**ene sequence analyses

At the C-terminus of the enniatin synthetase (Esyn) gene cluster two PCP domains are located, one catalyzing ester bonds and thereby performing peptide elongation and cyclisation, and another one acting as a waiting position^23^,^24^. A similar mechanism can be ruled out for cereulide synthesis, since gene sequence analysis did not reveal any evidence for duplicated PCP domains in CesA or CesB (data not shown). A second alternative might be that cereulide synthesis follows the classical collinear principle of NRPS product assembly including partial hydrolysis of the preformed tetradepsipetides, mediated by condensation domain reversibility. For instance, VibFC2 and VibH are not only mediating condensation in vibriobactin synthesis but also catalyze hydrolysis of single monomers by back reaction^25^. However, the latter mechanism is not supported by our experimental findings for two reasons: first, only didepsipeptides and a multiple thereof were found by means of UPLC-TOF MS and not even traces of depsipeptide intermediates showing uneven numbers of monomers were detectable; second, a controlled partial hydrolysis is contradictory to the observed occurrence of two (**DP6a/b**) instead of one hexadepsipeptide and two (**DP10a/b**) instead of one decadepsipeptide, respectively (Table 1).

**Figure 7:**
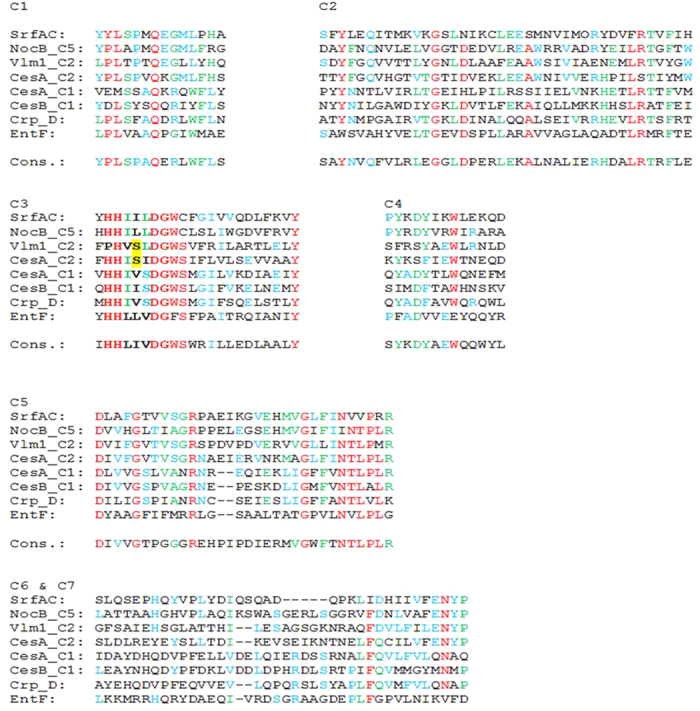
Alignment of the condensation (C) domain core motifs of Ces and core motifs from selected bacterial NRPS. SrfAC: terminal C domain from surfactin synthetase as representative of a DCL; NocB: C5 domain from the nocardicin synthetase as representative of a C domain with non-canonical functions; Vlm1: C2 domain in the valinomycin synthetase, which shows the same genetic architecture as the cereulide synthetase; Crp_D: C domain (M2) from cryptophycin synthetase catalyzing ester bound formation; EntF: C domain from enterobactin synthetase. According to literature^42^, the consensus sequence from Pfam database for core motifs C1 to C5 is depicted. The C3 core motif comprises the conserved catalytic motif HHxxxDG (printed in bold).

Besides their well-known pivotal role in peptide bound formation, C domains have been reported to catalyze ester bound formation and may also have dual functions by acting as epimerization and condensation domain^26^,^27^,^28^. Very recently, an unprecedented function of a C domain was reported for beta lactam antibiotics of the nocardicin family. The C5 domain of nocardicin NRPS (NocB) not only fulfills its canonical function by forming the corresponding amide bond in the growing peptide chain, but also mediates cyclization of the beta lactam ring^29^. Thus, the spectrum of additional functional activities reported for C domains in non-ribosomal multi enzyme complexes is still growing and may significantly contribute to the diversification of naturally synthetized peptide products. As shown in Fig. 7, primary sequence analysis of the Ces C domains revealed that CesB_C3 belongs to the L C domain class (LCL) while CesA_C2 belongs to the D C domain class (DCL)^30^, which is in full agreement with the stereochemistry of cereulide. CesA_C1 shows some characteristic features of LCL, albeit, based on the depsipeptide structure and previous stereochemical experiments, one would rather expect a DCL^8^. A similar deviation from the expected C class was reported for NocB^29^. The C5 domain of NocB shows DCL characteristics, despite receiving an L-peptide (Fig. 7). Comparison of Ces C domains to C domain sequences in the natural product domain seeker database NaPDoS^31^ revealed a distinct clustering of CesA_C1 and CesB_C3 while CesA_C2 was grouping with dual C domains and DCL (data not shown).

Remarkably, the core motifs of CesA_C2 show a relatively high homology to the second C domain in Vlm1 of the valinomycin NRPS although the overall similarities between the two NRPS is low (approx. 33%). Since formation of cereulide and valinomycin is thought to follow the same biosynthetic logic^8^, it is proposed that CesA_C2 and Vlm1_C2 act as ester synthases rather than as amide synthases. For this function, the location of the C domains, designated as C*, at the C terminus of CesA/Vlm1 might be of relevance. C domains are most commonly encoded in the same coding DNA sequence (CDS) as their acceptor molecules, upstream of the corresponding A domain, and there are only very few examples were a C domain is located in the C terminal proximitiy of the preceding CDS in NRPS^32^,^33^,^34^. For the C5 domain of NocB a unique amino acid located immediately upstream to the conserved HHxxxDG motif has been reported, which is crucial for the proper function of C5^29^. The HHxxxDG motif promotes the condensation reaction and transfer of the intermediate to the downstream PCP, with the second His thought to be central for the catalytic activity of the C domain^12^. Interestingly, we also observed an unusual amino acid inserted the HHxxxxDG motif of CesA_C2 and Vlm1_C2 (Fig. 7). Intensive database searches of NRPS C domains revealed a serine at a position in CesA_C2 (C*) and Vlm_C2 were usually aliphatic amino acids are positioned (Fig. 7). Further structural and biochemical analyses, which are clearly beyond the scope of this study, will be necessary in the future to fully decipher the role of C* in cereulide assembly. However, the location of C* in the C terminus of CesA may facilitate a postulated function of this domain as esterification center for cereulide assembly.

### Proposed biosynthesis of cereulide

**Figure 8:**
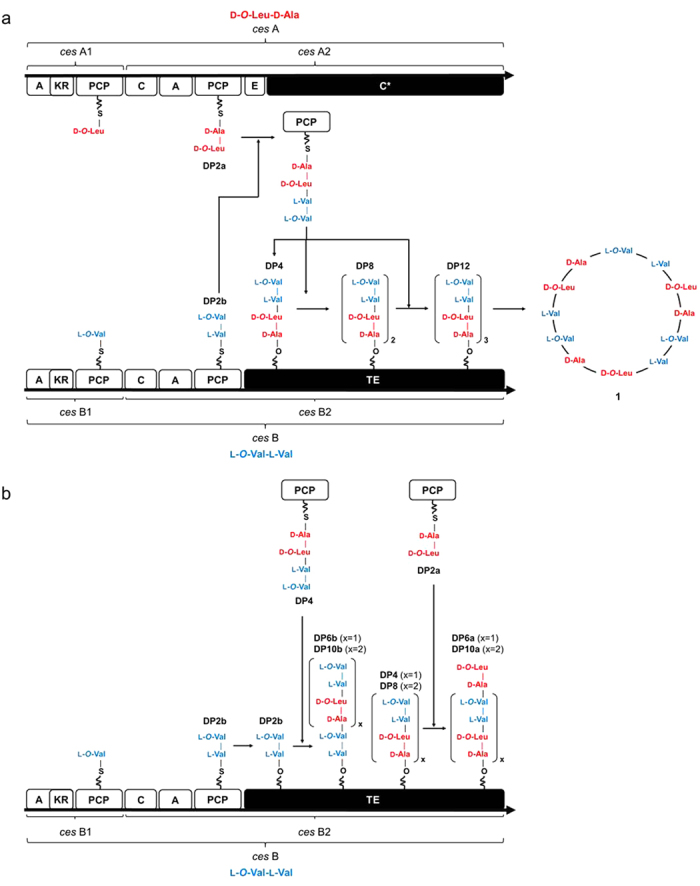
(**A**) Proposed biosynthesis of cereulide. The CesA and CesB modules of cereulide NRPS are made up the adenylation (**A**) domain, that recognize, select, and reduce α-ketocarboxylic acids to α-hydroxyacyl-*S*-pantetheinyl intermediates by means of a ketoreductase (KR) domain. The peptidyl carrier protein (PCP) captures the precursor adenylates as covalent thioesters via a phosphopantetheine linker. Dipeptide formation is catalyzed by a condensation (**C**) domain catalyzing the nucleophilic attack of the downstream PCP-bound acceptor monomer on the activated thioester of the upstream PCP-bound donor monomer intermediate, thus D-*O*-Leu-D-Ala (**DP2a**) and L-*O*-Val-L-Val (**DP2b**) are generated independently by CesA and CesB. The C* domain catalyzes the ester bond formation to reveal the PCP-bound tetradepsipeptide **DP4** which is subsequently transferred to the CesB C-terminal TE domain. Again catalyzed by the C* domain, a second **DP4** is then connected to the TE-bound tetradepsipeptide by esterification, thus affording **DP8** and **DP12** upon extension by a third **DP4** unit prior to macrolactonization to cereulide (**1**). (**B**) As side products, hexa- and decadepsipeptide esters are generated by an incorrect transfer of **DP2a** onto **DP4** and **DP8** leading to the **DP6a** and **DP10a**, respectively. Transesterification of **DP2b** onto the TE domain, followed by C* domain catalyzed esterification with one or two units of **DP4** gives rise to the **DP6b** and **DP10b**, respectively.

Taking all the experimental data and bioinformatic analysis on the *ces* gene locus into account and considering previously published data^8^,^9^, the following modified mechanism for the biosynthesis of cereulide is proposed (Fig. 8a). Initially, the dipeptides D-*O*-Leu-D-Ala and L-*O*-Val-L-Val, both bound as a thioester at PCP, are generated independently by CesA and CesB. The proposed condensation domain C* (CesA_C2)^8^ transfers the hydroxy group of D-*O*-Leu-D-Ala-*S*-PCP to the thioester carbon of L-*O*-Val-L-Val-*S*-PCP to form an ester bond resulting in the PCP-bound tetradepsipeptide L-*O*-Val-L-Val-D-*O*-Leu-D-Ala-*S*-PCP (**DP4**)^24^, which is subsequently transferred to the serine hydroxy group of the CesB C-terminal TE domain (Fig. 8a). Catalyzed by the condensation domain C* (CesA_C2), a second L-*O*-Val-L-Val-D-*O*-Leu-D-Ala-*S*-PCP, produced as described above, is then connected to the TE-bound tetradepsipeptide by esterification, thus forming the octadepsipeptidyl-*O*-TE intermediate (**DP8**) and, further on, the dodecadepsipeptide-*O*-TE (**DP12**) upon extension by a third L-*O*-Val-L-Val-D-*O*-Leu-D-Ala unit prior to macrolactonization to the cyclic twelve residue product cereulide (Fig. 8a). It is therefore proposed that the C* domain located at the C-terminus of CesA and the TE domain located at the C-terminus of CesB function as a cooperative esterification and depsipeptide elongation center (**DP4** → **DP8** → **DP12**), repeatedly recruiting the action of the C* domain prior to macrocyclization by the TE domain. It is tempting to speculate that a previously described internal promoter of yet unknown function may play a role in adjusting and fine tuning of the independent transcription of *cesA* and *cesB,* thereby decoupling CesA and CesB to a certain extend^11^. Alternative modular expression of the polyketide synthetase pikAIV has been reported to be involved in the generation of two macrolactones differing in size while a potential contribution of alternative gene expression to the structural diversification of NRPS products still needs to be deciphered^35^.

Although NRPS product assembly frequently seems to display a strict correlation between the enzymatic domain sequence of the megasynthase and the position of the amino acid building blocks in the peptide product, this colinearity rule is not strictly reinforced in nature^36^,^37^. Recently, the molecular mechanisms involved in the noncanonical biosynthesis of the thalassospiramide family of cyclic lipopeptides have been convincingly demonstrated to involve atypical NRPS biochemical features like module skipping and multimodule iteration^38^.

The proposed mechanism considering dipeptides rather than single monomers as the basic modules in tetradepsipeptide assembly and oligomerization of tetradepsipeptides to give octa- and dodecadepsipeptides is well in line with the higher abundance of tetra-, octa-, and dodecadepsipeptide esters released upon ethanolysis when compared to di-, hexa-, and decadepsipeptide esters (Fig. 6). The low abundance hexa- and decadepsipeptide esters are proposed to be generated by an incorrect transfer of D-*O*-Leu-D-Ala-*S*-PCP (**DP2a**) onto L-*O*-Val-L-Val-D-*O*-Leu-D-Ala-*O*-TE (**DP4**) and (L-*O*-Val-L-Val-D-*O*-Leu-D-Ala)_2_-*O*-TE (**DP8**) leading to the generation of the hexadepsipeptide *O*-Leu-D-Ala-(L-*O*-Val-L-Val-D-*O*-Leu-D-Ala)-*O*-TE (**DP6a**) and *O*-Leu-D-Ala-(L-*O*-Val-L-Val-D-*O*-Leu-D-Ala)_2_-*O*-TE (**DP10a**), respectively (Fig. 8b). Incorrect transesterification of L-*O*-Val-L-Val-*S*-PCP (**DP2b**) to the TE domain, followed by C* domain catalyzed esterification with one or two units of tetradepsipeptidyl-S-PCP gives rise to the TE-bound hexadepsipeptide (L-*O*-Val-L-Val-D-*O*-Leu-D-Ala)-L-*O*-Val-L-Val-*O*-TE (**DP6b**) and decadepsipeptide (L-*O*-Val-L-Val-D-*O*-Leu-D-Ala)_2_-L-*O*-Val-L-Val-*O*-TE (**DP10b**), respectively.

These newly proposed mechanisms also help to explain the biosynthesis of isocereulides A (**2**) to G (**8**). Formation of hexa- and decadepsipeptides via NRPS misoperation is key to form the isocereulides E, F, and G, whereas peptide assembly in the biosynthesis of isocereulides A-D corresponds well to that of cereulide differing that L-*O*-Val is substituted by L-*O*-Leu (isocereulide A), L-Val by L-Ala (isocereulide D), D-Ala by D-Ser (isocereulide C), and D-O-Leu by D-O-Val (isocereulide B) due to a relaxed substrate specificity of the A domains and/or an inefficient proof reading by CesT.

In summary, this study reveals novel insights into the biosynthesis of cereulide and isocereulides, highlighting dipeptides rather than single amino or hydroxy acids as the basic modules in tetradepsipeptide assembly. It is proposed that the C* domain located at the C-terminus of CesAand the TE domain located at the C-terminus of CesB works as a cooperative esterification and depsipeptide elongation center, repeatedly recruiting the action of the C* domain to oligomerize tetradepsipeptides to give octa- and dodecadepsipeptides prior to macrocyclization by the TE domain. Since the valinomycin synthetase Vlm shows the same genetic architecture as Ces, it is tempting to speculate that the novel mechanism of non-ribosomal peptide assembly proposed in this study is not a specificity of the cereulide synthetase but a more common logic for biosynthesis of ester bond containing NRPS products, thereby contributing to biodiversity of natural depsipeptide products. Further structural and functional studies will be necessary to decipher the exact mechanism of the unusual C* domain located in *trans* in the C-terminus of CesA. Such knowledge would not only contribute to a better understanding of the complex biosynthetic pathways involved in the natural generation of depsipeptides, but could also open new avenues for the development of novel pharmaceuticals.

## Methods

### Chemicals

The following chemicals were purchased for cultivation and extraction of bacterial cultures: MeOH and EtOH (Mallinckrodt Baker B.V., Deventer, Holland), pepton tryptone, yeast extract (Oxoid Hamphsire, England), NaCl (Carl Roth, Karlsruhe, Germany), and D-(+)-glucose monohydrate (Fluka, Sigma Aldrich, Steinheim, Germany). H_2_O for chromatography was purified with an integral-5 system (Millipore, Schwalbach, Germany), solvents used were of HPLC or LC-MS grade (J.T. Baker, Deventer, Holland). Purified reference material of cereulide (**1**) and isocereulide A-G (**2**–**8**) was prepared as reported recently^17^,^18^. The dipeptides D-*O*-Leu-D-Ala and L-*O*-Val-L-Val were synthesized following a literature procedure^18^. Cultivation of strain F4810/72 in MOD-medium supplemented with ^13^C_1_-valine was performed as reported recently^18^,^19^.

### Bacterial Strains and Growth Conditions

Five *B. cereus* strains were used in this study: the emetic reference strain F4810/72 and the foodborne intoxication strain WSBC 10925^39^, both recently classified as a cereulide high producers, and the cereulide low to medium producers F3876/87 isolated from a patient’s vomit and E07395/2 and WSBC 10942, both isolated from *B. cereus* intoxicated foods^17^. LB broth (100 mL) supplemented with D-glucose (0.2%) was inoculated with an overnight pre-culture (10^3^ cfu/mL) and, then, incubated at 24 °C in baffled flasks (500 mL) whilst rotary shaking (150 rpm). After incubation for 24 h, the culture of strain F3876/87 was autoclaved (15 min, 121 °C) and centrifuged (7800 × g, 121 °C, 12 min; Sigma 3-18 K), pellets from culture portions (100 mL) were frozen in liquid nitrogen and stored at -20 °C until further use.

### Comparative UPLC-TOF MS Profiling of *B. cereus* Strains WSBC 10925 and F3876/87

Bacterial pellets, harvested from cultures (100 mL) of strains WSBC 10925 and F3876/87, respectively, were extracted with EtOH (10 mL) by shaking at room temperature for 15 h, the extract was centrifuged twice (7800 × g, 20 °C, 12 min), the supernatant was centrifuged (18600 × g, 20 °C, 5 min) and membrane filtered (0.2 *μ*m; Phenomenex, Aschaffenburg, Germany) to remove remaining cells and cell debris. Aliquots (3 *μ*L) of ethanolic cell pellet extracts were analysed by means of UPLC-TOF MS using the following solvent gradient for chromatography (0.4 mL/min, 45 °C): starting with a mixture (10/90, v/v) of water and methanol, the methanol content was increased to 100% within 8 min and, then, kept constant for 1 min.

### Mass Spectrometric Screening of Solvent Extractables from Strain F3876/87

Bacterial pellets, prepared from F3876/87 liquid cultures (100 mL) by centrifugation, were extracted with 10 mL of MeOH, MeCN, or MeCN/H_2_O (70/30, v/v), respectively, by shaking overnight (15 h) at room temperature. The individual solvent extracts were centrifuged (7800 × g; 20 °C, 12 min), membrane-filtered (0.2 *μ*m; PTFE; Phenomenex, Aschaffenburg, Germany) to remove remaining cell debris, made up to 10 mL with MeOH and, then, analyzed by means of UPLC-TOF MS using the following chromatographic conditions (0.4 mL/min, 40 °C): starting with a mixture of H_2_O/MeOH (10/90, v/v), the MeOH content was kept constant for 1 min at 90%, increased to 100% within 8 min, kept constant for 1 min, decreased within 0.1 min to 90% MeOH, followed by re-equilibration for 0.4 min.

### Isolation and Structure Determination of a Candidate Tetradepsipeptide Ethyl Ester (*m/z* 453) and Octadepsipeptide Ethyl Ester (*m/z* 837), Respectively

The combined EtOH extracts obtained from cultures (100 mL) of strain F4810/72 were concentrated to about 25 mL in vacuum and, after dilution with H_2_O (1/10, v/v), aliquots (5 mL) were separated by means of preparative RP-HPLC on a PrepStar system (Varian, Darmstadt, Germany) consisting of two HPLC-pumps (Model SD-1), a two-wavelength UV detector (Prostar 325), a fraction collector (Model 701), and equipped with a 250 × 21.2 mm, 4 *μ*m, 90A, Jupiter Proteo column (Phenomenex) as the stationary phase. Monitoring the effluent at 220 nm, chromatography (flow rate: 18.0 mL/min) was performed starting with a MeOH/H_2_O mixture (85/15, v/v) for 1 min, increasing the MeOH content to 100% within 10 min, holding for 10 min, followed by a decrease of the MeOH content to 85% within 1 min and re-equilibration for 2 min at 85% MeOH. A total of 14 HPLC fractions were collected, separated from solvent in vacuum, the residue of each fraction was suspended in H_2_O (10 mL), freeze-dried twice and, then, screened for the target compounds showing the pseudomolecular ions *m/z* 453 and 837, respectively, by means of UPLC-TOF MS using the following chromatographic conditions (0.3 mL/min, 40 °C): starting with a mixture of H_2_O/MeOH (40/60, v/v), the MeOH content was kept constant for 1 min at 60%, increased to 90% within 8 min, increased to 95% within 10 min, increased to 100% within 1 min, kept constant for 1 min, decreased within 1 min to 60% MeOH, followed by re-equilibration for 1 min. UPLC-TOF MS analyses revealed the candidate tetradepsipeptide ethyl ester (*m/z* 453) and the octadepsipeptide ethyl ester (*m/z* 837) in HPLC fraction 3.

Further purification was done by means of analytical HPLC on a 250 × 4.6 mm, 4 *μ*m, 90 A, Jupiter Proteo column (Phenomenex). Monitoring the effluent at 220 nm, chromatography (flow rate: 1.0 mL/min) was started with a mixture of H_2_O/MeOH (40/60, v/v), the MeOH content was kept constant for 1 min at 60%, increased within 20 min to 80%, increased within 5 min to 86%, increased within 10 min to 90%, increased within 1 min to 100%, kept constant for 3 min, decreased within 1 min to 60% MeOH, followed by re-equilibration for 4 min. After removing the solvent in vacuum, the candidate tetradepsipeptide ethyl ester (*m/z* 453; 0.4 mg) and the octadepsipeptide ethyl ester (*m/z* 837; 0.3 mg) obtained were suspended in water (3 mL) and freeze-dried. Aliquots (~0.1 mg) of the target compounds were dissolved in methanolic KOH solution (1.2 mol/L, 80% MeOH), incubated at 50 °C for 2 h, then adjusted to pH 5.0, and analyzed by means of UPLC-TOF MS in the negative electrospray mode. Chromatography was performed on a 2.1 × 150 mm, 1.7 *μ*m, BEH C18 column (Waters) at 45 °C using aqueous HCOOH (0.1% in H_2_O) as solvent A and MeCN containing 0.1% HCOOH as solvent B and the following gradient (flow rate: 0.4 mL/min): For dipeptide analysis in the hydrolysates, chromatography started with 1% solvent B for 2.5 min, which was increased to 95% within 5.5 min, hold for 1.5 min and, then, decreased again to 1% within 0.5 min. For TOF MS analysis, scan time for the MS^e^ method (centroid) was set to 0.1 sec, high-resolution negative ionization mode and the following ion source parameters were selected: capillary voltage (-2.5 kV), sampling cone (50 V), source temperature (150 °C), desolvation temperature (450 °C), cone gas (10 L/h) and desolvation gas (850 L/h). For the MS^e^ method, the second MS scan function used a transfer collision energy ramp from 20 to 40 eV.

### Incubation of *B. Cereus* in the Presence of ^13^C_6_-Cereulide

Aliquots (19.8 mL) of LB medium (including 0.2% glucose) were mixed with an ethanolic solution (0.2 mL) of ^13^C_6_-cereulide (10 *μ*g/mL) and, then, inoculated with the *B. cereus* strain F4801/72 (10^3^ KBE/mL). Before (time point: 0 h) and after incubation for 12, 20, and 40 h at 24 °C whilst shaking (150 rpm), aliquots (2.0 mL) were taken, autoclaved (15 min, 121 °C), pellets collected by centrifugation (13000 rpm, 4 min), and extracted with EtOH (1 mL) by shaking overnight (15 h) at room temperature. After centrifugation (7800 × g; 12 min, each), the supernatants were membrane-filtered (0.2 *μ*m; PTFE; Phenomenex), made up to 1 mL with EtOH and, then, directly used for UPLC-TOF-MS analysis.

### Mass Spectrometry

High-resolution mass spectrometric analyses (UPLC-ESI-TOF MS) were performed on a Waters Synapt G2-S HDMS spectrometer coupled to an Acquity UPLC core system (Waters, Milford, MA, USA) consisting of a binary solvent manager, sample manager and a 2 × 150 mm, 1.8 *μ*m, HSS T3 C18 column (Waters, Manchester, UK). Data processing was performed by using MassLynx 4.1 SCN 851 (Waters, Manchester, UK) and the elemental composition tool for determining the accurate mass. MS analyses were performed in the positive ESI and high resolution mode with the instrument setting for the capillary voltage (+2.5 kV), sampling cone (50 V), source temperature (120 °C), desolvation temperature (450 °C), cone gas (10 L/h), and desolvation gas (850 L/h) given in parenthesis. All data were lock mass corrected on the pentapeptide leucine enkephaline (Tyr-Gly-Gly-Phe-Leu, *m/z* 556.2771, [M+H]^+^ and *m/z* 554.2615, [M-H]^–^) in a solution (2 ng/*μ*L) of MeCN/0.1% HCO_2_H (1/1, v/v). Scan time for the lock mass was set to 0.3 s, an interval of 10 s and 3 scans to average with a mass window of ±0.5 Da. Calibration of the Synapt G2-S in the range from *m/z* 50 to 1300 was performed using a solution of sodium formate (5 mmol/L) in 2-propanol/H_2_O (9/1, v/v). The UPLC and Synapt G2-S systems were operated with MassLynx software (Waters, Manchester), data processing and analysis were performed using MarkerLynx XS^TM^ software. The raw data obtained from UPLC-TOF MS analysis were processed with MarkerLynx XS using ApexTrack peak integration to detect chromatographic peaks. Marker intensity threshold was set to 2000 cps, mass window was 0.02 Da, retention time window was 0.1 s, and data were de-isotoped.

MS^n^-experiments were performed on a Bruker Daltonics HCTultra PTM Discovery System^TM^ (Bruker Daltonics Inc., Billerica, MA, USA) using direct sample infusion via a syringe pump (4 *μ*L/min; kd scientific, USA) and manual MS^n^ (*m/z* 50-1300) using the following instrument parameters: ultra-scan mode (26.000 *m/z*/sec), max. accumulation time (300 ms), isolation width (*m/z* 4), collision amplitude (1 V; ramp: 30-200%). The following source parameters were applied: capillary voltage (+4 kV), end plate offset (-500 V), nebulizer (30 psi), dry gas (8 L/min), dry temperature (300 °C), skimmer (40 V), capillary exit (166 V). Data acquisition and processing were done by using Bruker Daltonics Data Analysis Version 3.4 (Bruker Daltonics Inc.).

### Bioinformatic Analysis of the Cereulides Synthetase Gene Locus

The previously sequenced cereulide synthetase gene locus *ces*^9^ was derived from the NCBI database (Acc.No. NG_036207) and subjected to bioinformatics analysis. Sequence similarity searches were performed using the Basic Local Aligment Search Tools BLASTP^40^. The software packages ClustalX and TREECON were used for sequence alignments and cluster analysis^41^,^42^. To search for conserved sequence domains the online software tool Pfam^43^ was employed and analysis of the condensation domain function was carried out using the database NaPDos^31^. The C domain core motifs were identified according to Rausch *et al.*^30^

## Additional Information

**How to cite this article**: MARXEN, SANDRA *et al.* Depsipeptide Intermediates Interrogate Proposed Biosynthesis of Cereulide, the Emetic Toxin of Bacillus cereus. *Sci. Rep.* **5**, 10637; doi: 10.1038/srep10637 (2015).

## Supplementary Material

Supporting Information

## Figures and Tables

**Figure 1:**
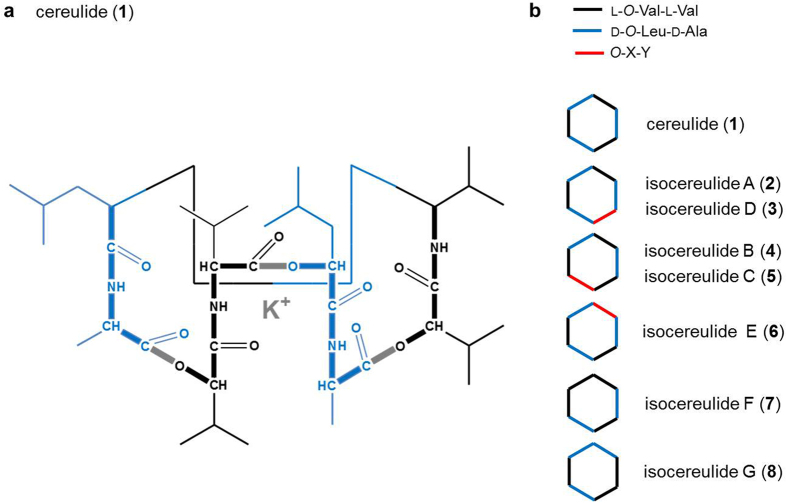
(**a**) Chemical structure of the cyclic dodecadepsipeptide cereulide (**1**), [D-*O*-Leu-D-Ala-L-*O*-Val-L-Val]_3_, (**b**) chemical structure of isocereulides A (**2**) to G (**8**), *O*-X-Y indicates the position of the dipeptides L-*O*-Leu-L-Val (isocereulide A ), D-*O*-Val-D-Ala (isocereulide B), D-*O*-Leu-D-Ser (isocereulide C), L-*O*-Val-L-Ala (isocereulide D), D-*O*-Ile-D-Ala (isocereulide E).

**Table 1:** Chromatographic and Mass Spectrometric Data Oligodepsipeptides (**DP2a/b** – **DP12**) and their Methyl and Ethyl esters Released from *B. cereus* Cultures upon Extraction with MeCN/H_2_O (70/30, v/v), MeOH, and EtOH, Respectively.

**No.**^**a**^	**Peptides (R=H) and Peptide Esters (R=Me, Et)**^**b**^	**UPLC-TOF MS data**							
		**free peptides**^**c**^		**methyl ester**^**d**^		**ethyl ester**^**e**^		^**13**^**C-labeled ethyl ester**^**f**^	
		**RT**^**g**^**(min)**	**EC**^**h**^ **(EM/AM; var., ppm)**^**i**^	**RT**^**g**^ **(min)**	**EC**^**h**^ **(EM/AM; var., ppm)**^**i**^	**RT**^**g**^**(min)**	**EC**^**h**^ **(EM/AM**^**h**^**; var., ppm)**^**i**^	**RT**^**g**^**(min)**	**EC**^**h**^ **(EM/AM**^**h**^**; var., ppm)**^**i**^
**DP2a**	D-O-Leu-D-Ala-O-R	0.97	C_9_H_17_NO_4_Na (226.1055/226.1055; -0.1)	1.00	C_10_H_19_NO_4_Na (240.1212/240.1211; +0.1)	1.05	C_11_H_21_NO_4_Na (254.1368/254.1377; +3.5)	—	—
**DP2b**	L-O-Val-L-Val-O-R	0.99	C_10_H_19_NO_4_Na (240.1212/240.1211; -0.1)	1.05	C_11_H_21_NO_4_Na (254.1368/254.1373; +2.0)	1.12	C_12_H_23_NO_4_Na (268.1525/268.1537; +4.5)	—	—
**DP4**	L-O-Val-L-Val-D-O-Leu-D-Ala-O-R	1.10	C_19_H_34_N_2_O_7_Na (425.2264/425.2265; +0.2)	1.17	C_20_H_36_N_2_O_7_Na (439.2420/439.2420; -0.1)	1.24	C_21_H_38_N_2_O_7_Na (453.2577/453.2569; -1.8)	1.24	^12^C_19_^13^C_2_H_38_N_2_O_7_Na (455.2644/455.2645; -1.8)
**DP6a**	D-O-Leu-D-Ala-(L-O-Val-L-Val-D-O-Leu-D-Ala)-O-R	1.14	C_28_H_49_N_3_O_10_Na (610.3316/610.3323; +1.1)	1.28	C_29_H_51_N_3_O_10_Na (624.3472/624.3477; +0.8)	1.35	C_30_H_53_N_3_O_10_Na (638.3629/638.3628; -0.2)	1.35	^12^C_28_^13^C_2_H_53_N_3_O_10_Na (640.3696/640.3688; -1.2)
**DP6b**	(L-O-Val-L-Val-D-O-Leu-D-Ala)-L-O-Val-L-Val-O-R	1.22	C_29_H_51_N_3_O_10_Na (624.3472/624.3472; -0.3)	1.45	C_30_H_53_N_3_O_10_Na (638.3629/638.3635; +0.9)	1.52	C_31_H_55_N_3_O_10_Na (652.3785/652.3777; -1.2)	1.52	^12^C_27_^13^C_4_H_55_N_3_O_10_Na (656.3919/656.3916; -0.5)
**DP8**	(L-O-Val-L-Val-D-O-Leu-D-Ala)_2_-O-R	1.43	C_38_H_66_N_4_O_13_Na (809.4524/809.4530; +0.7)	1.75	C_39_H_68_N_4_O_13_Na (823.4681/823.4688; +0.9)	1.78	C_40_H_70_N_4_O_13_Na (837.4837/837.4798; -4.7)	1.78	^12^C_36_^13^C_4_H_70_N_4_O_13_Na (841.4971/841.4961; -1.2)
**DP10a**	D-O-Leu-D-Ala-(L-O-Val-L-Val-D-O-Leu-D-Ala)_2_-OR^j^	1.44	C_47_H_81_N_5_O_16_Na (994.5576/994.5580; +0.4)	1.97	C_48_H_83_N_5_O_16_Na (1008.5733/1008.5740; +0.7)	1.95	C_49_H_85_N_5_O_16_Na (1022.5889/1022.5871; -1.8)	1.94	^12^C_45_^13^C_4_H_85_N_5_O_16_Na (1026.6023/1026.6012; -1.1)
**DP10b**	(L-O-Val-L-Val-D-O-Leu-D-Ala)_2_-L-O-Val-L-Val-O-R	1.54	C_48_H_83_N_5_O_16_Na (1008.5733/1008.5735; +0.2)	2.32	C_49_H_85_N_5_O_16_Na (1022.5889/1022.5897; +0.8)	2.26	C_50_H_87_N_5_O_16_Na (1036.6046/1036.6031; -1.4)	2.21	^12^C_44_^13^C_6_H_87_N_5_O_16_Na (1042.6247/1042.6244; -0.3)
**DP12**	(L-O-Val-L-Val-D-O-Leu-D-Ala)_3_-O-R	1.78	C_57_H_98_N_6_O_19_Na (1193.6784/1193.6787; +0.3)	2.99	C_58_H_100_N_6_O_19_Na (1207.6941/1207.6953; +1.0)	2.71	C_59_H_102_N_6_O_19_Na (1221.7097/1221.7050; -3.8)	2.68	^12^C_53_^13^C_6_H_102_N_6_O_19_Na (1227.7299/1227.726; -3.1)

^a^Compound number of peptides detected; ^b^Sequence of open-chain peptides and peptide esters determined my MS^n^ analysis; ^c,d,e^ Corresponding peptides and esters released upon treatment of *B. cereus* cell pellets with MeCN/H_2_O (70/30, v/v), MeOH, and EtOH, respectively; ^f 13^C-Labeled ethyl esters released of strain F4810/72 cultivated in MOD-medium supplemented with ^13^C_1_-valine;^17^,^19^ ^g^ Retention time on RP-18 UPLC; ^h^ Elemental composition of analyte; ^i^ Exact mass (EM, calcd) and accurate mass (AM) of analyte’s [M+Na]^+^ ion determined by means of TOF MS.
