# Supplementary material for: Depsipeptide Intermediates Interrogate Proposed Biosynthesis of Cereulide, the Emetic Toxin of Bacillus cereus
Source: Sci Rep. 2015 May 27;5:10637. doi: 10.1038/srep10637 (PMC4445039; doi:10.1038/srep10637)

Supplementary Information

Depsipeptide Intermediates Interrogate Proposed Biosynthesis of Cereulide, the Emetic Toxin of *Bacillus cereus*

SANDRA MARXEN,[1] TIMO D. STARK,[1] ANDREA RÜTSCHLE,[2] GENIA LÜCKING,[2] ELRIKE FRENZEL,[3] SIEGFRIED SCHERER[2,4], MONIKA EHLING-SCHULZ,[3] AND THOMAS HOFMANN[1*]

Figure S1 Sequential MS^n-spectra of the ethyl esters of (**a**) tetradepsipeptide (**DP4**), (**b**) hexadepsipeptide (**DP6a**), (**c**) hexadepsipeptide (**DP6b**), (**d**) octadepsipeptide (**DP8**), (**e**) decadepsipeptide (**DP10a**), (**f**) decadepsipepide (**DP10b**), and (**g**) dodecadepsipeptide (**DP12**) (upper masses: calculated, lower masses given in parenthesis: measured ($[M+K]^+$) and ($[M+Na]^+$)* ions), ●: ^{13}C-labeling of α-hydroxy isovaleric acid and valine.

a) Tetradepsipeptide (**DP4**)

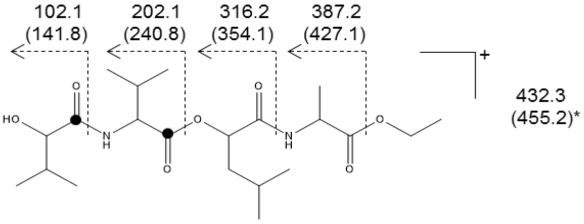

MS

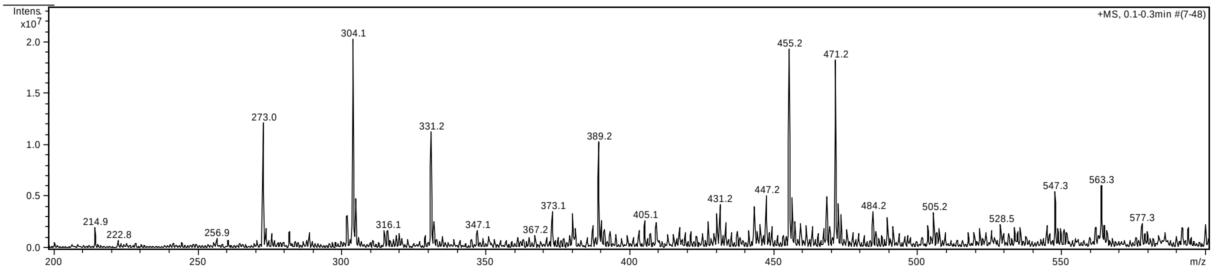

MS^2 (455.2 → x)

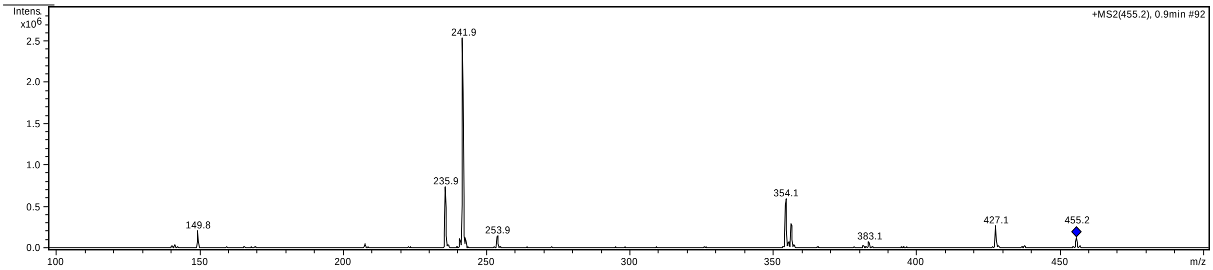

MS^3 (354.1 → x)

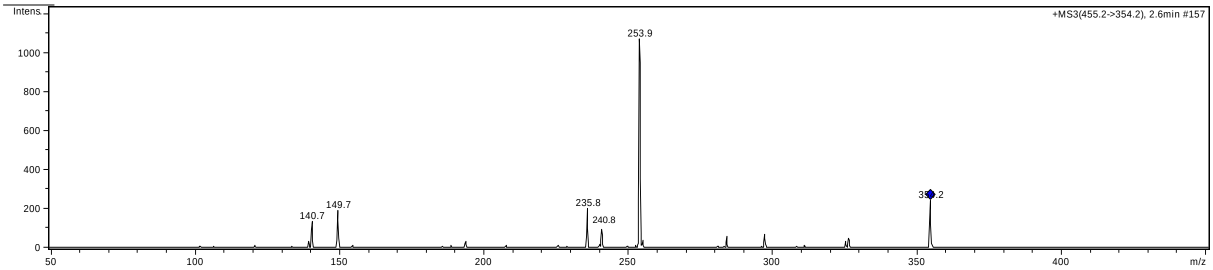

MS^4 (240.8 → x)

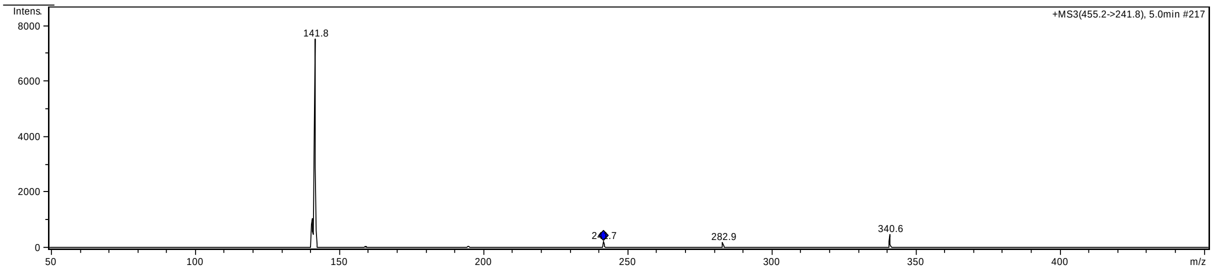

b) Hexadepsipeptide (**DP6a**)

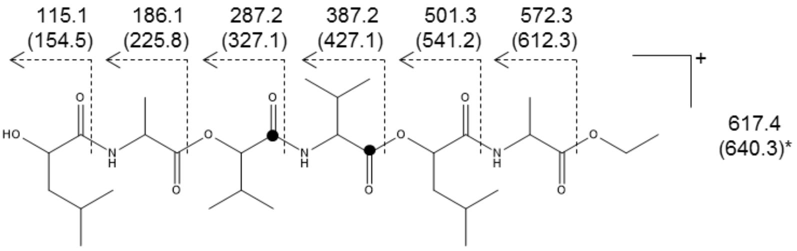

MS

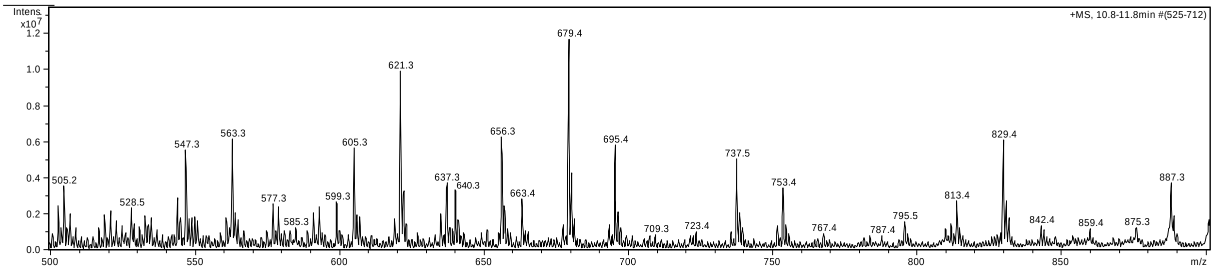

MS^2 (640.3 → x)

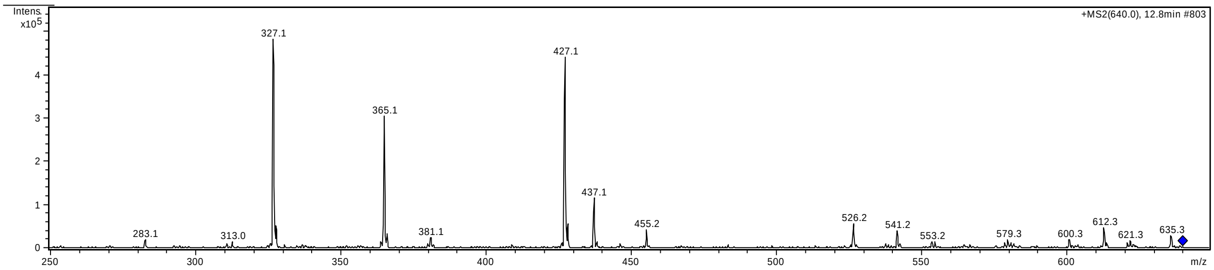

MS^3 (427.1 → x)

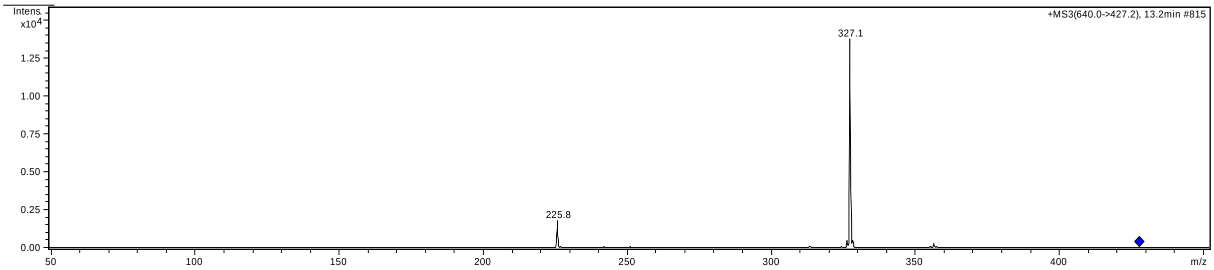

MS^4 (327.1 → x)

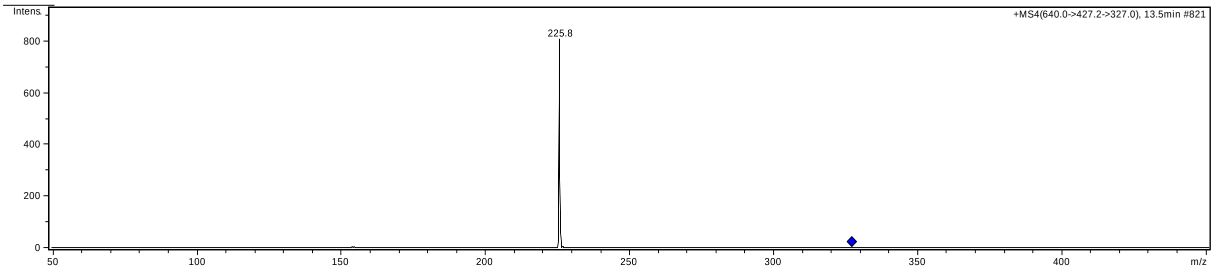

MS^4 (225.8 → x)

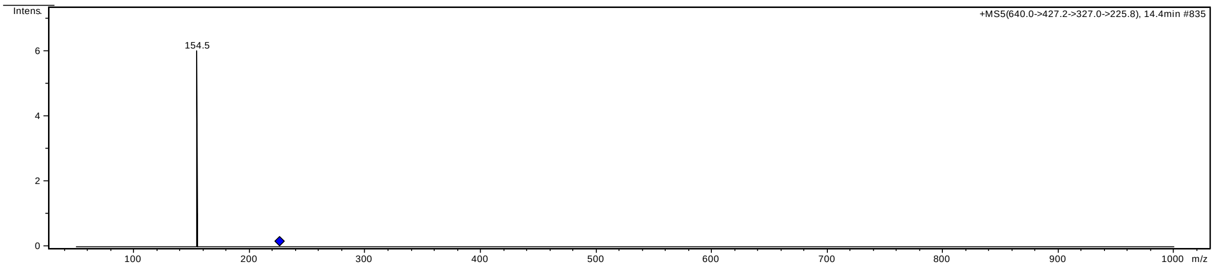

c) Hexadepsipeptide (**DP6b**)

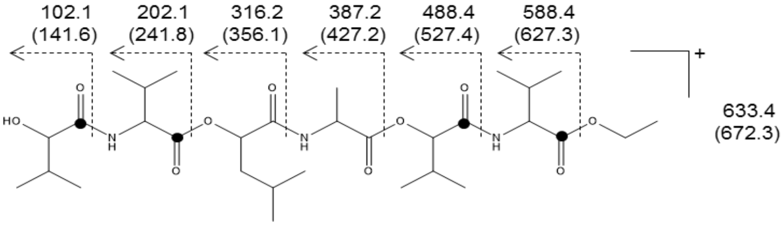

MS

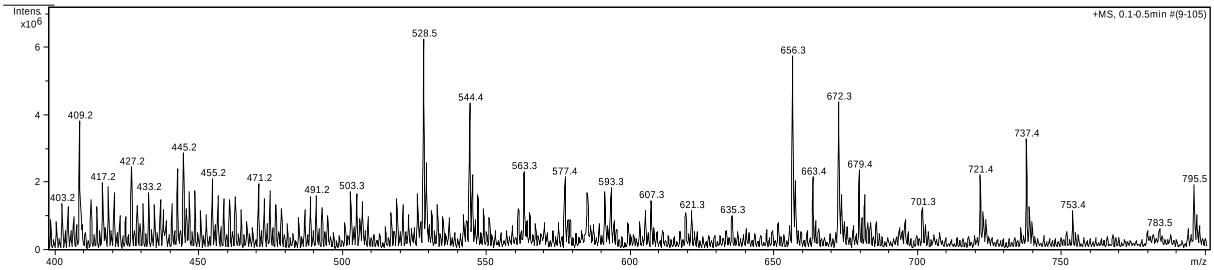

MS^2 (672.3 → x)

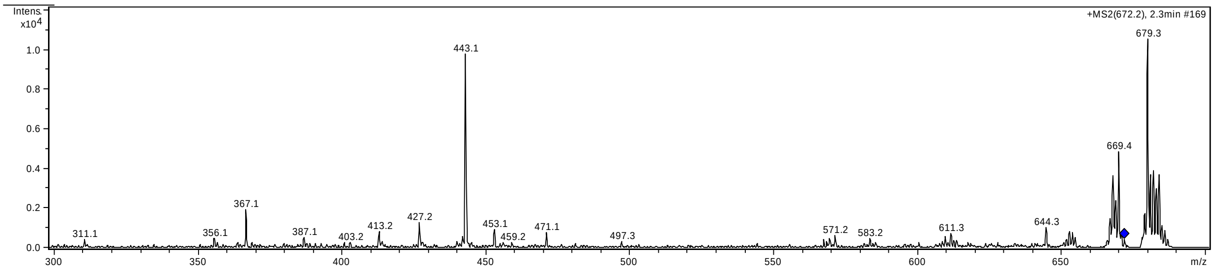

MS^3 (427.2 → x)

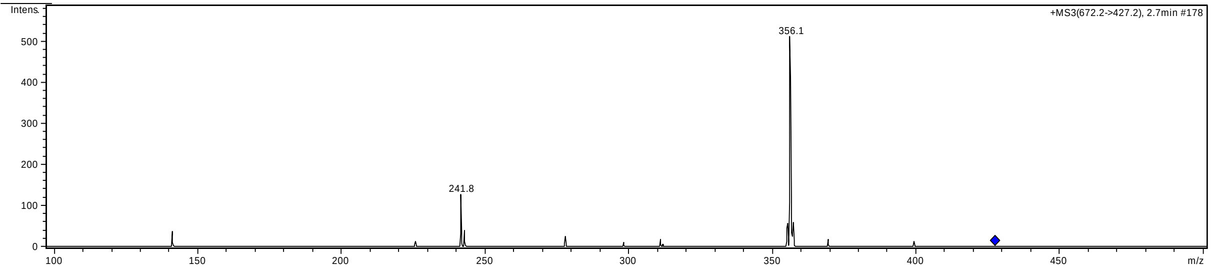

MS^4 (356.1 → x)

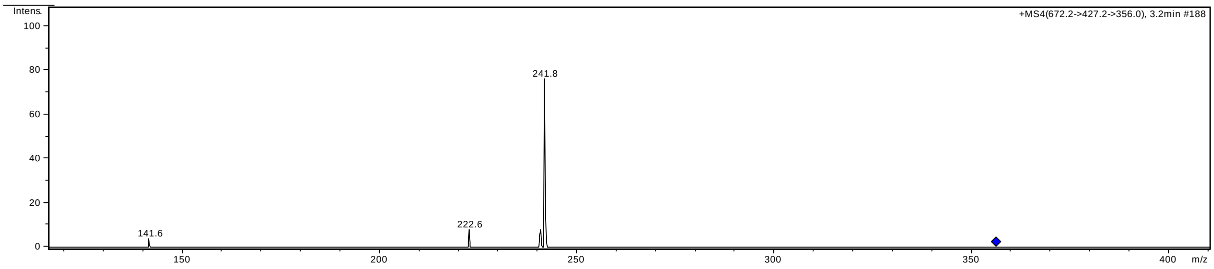

d) Octadepsipeptide (**DP8**)

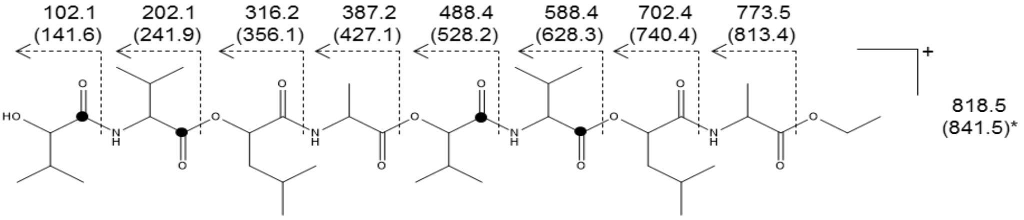

MS

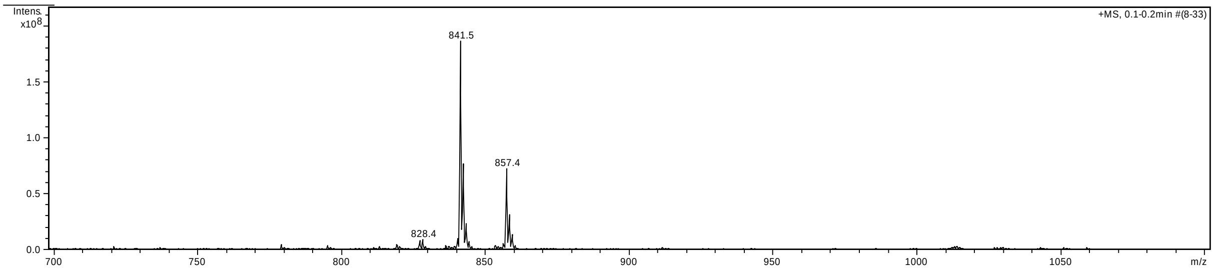

MS^2 (841.5 → x)

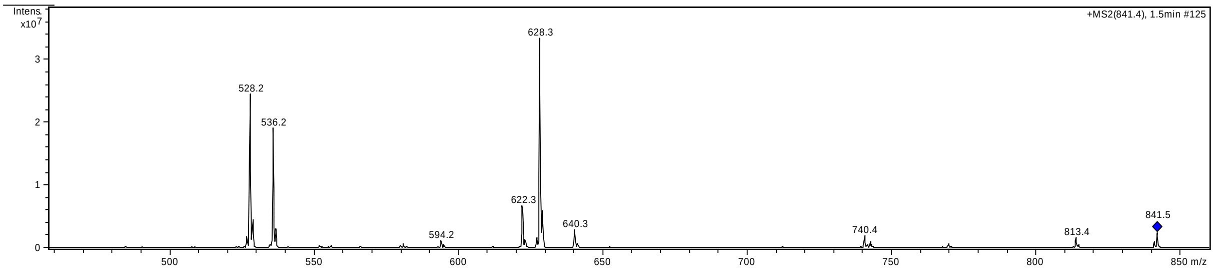

MS^3 (628.3 → x)

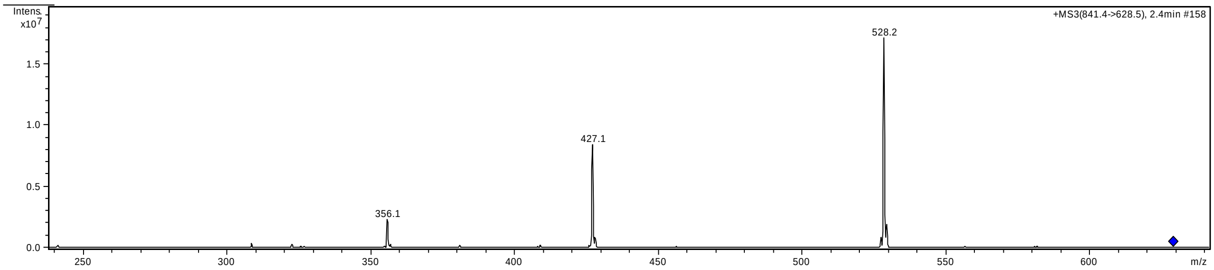

MS^4 (528.2 → x)

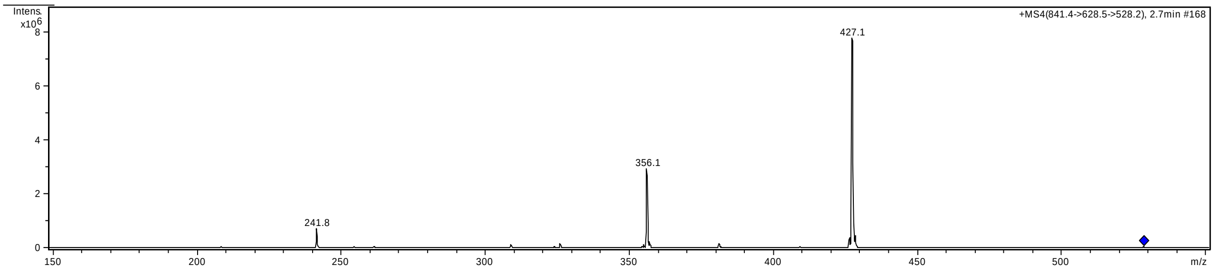

MS^5 (427.1 → x)

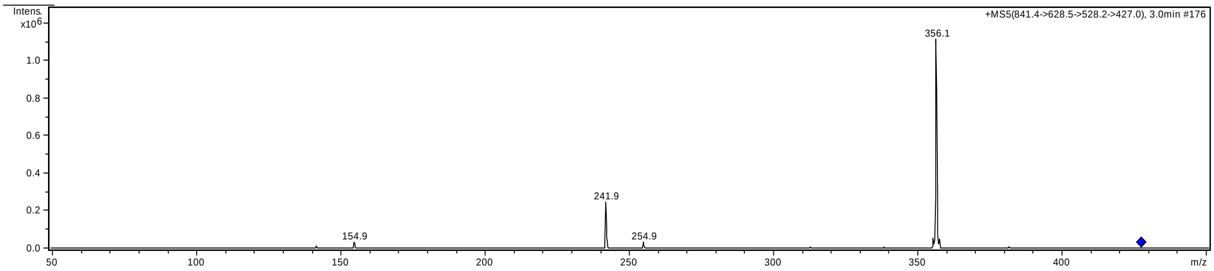

MS^6 (356.1 → x)

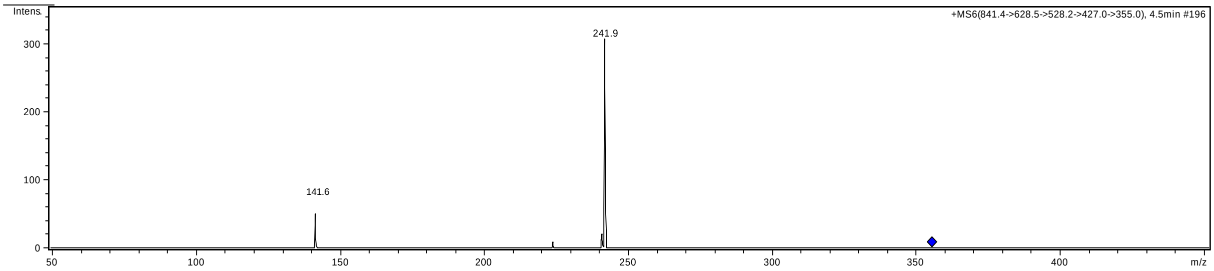

e) Decadepsipeptide (**DP10a**)

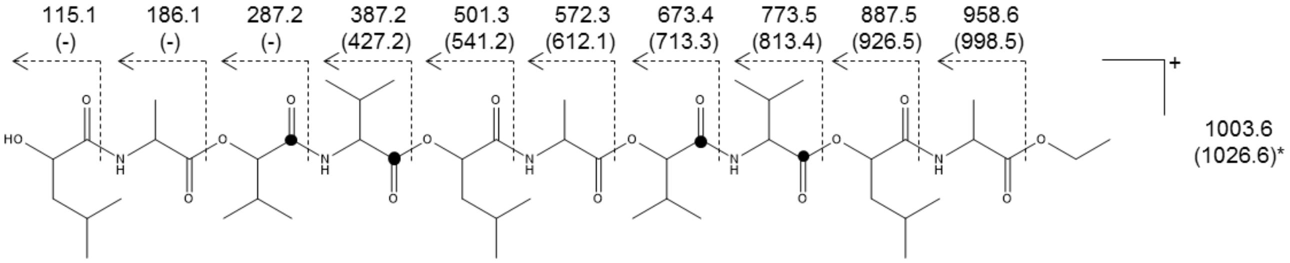

MS

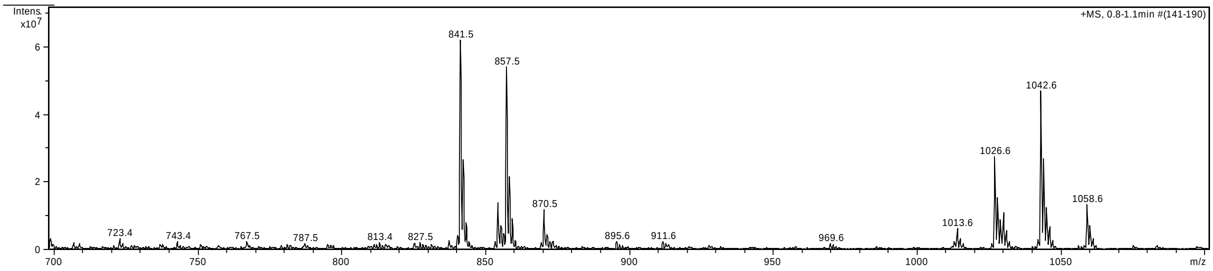

MS^2 (1026.6 → x)

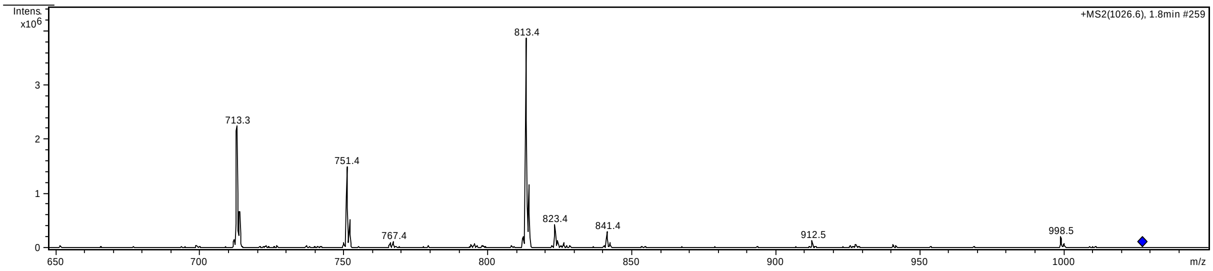

MS^3 (813.4 → x)

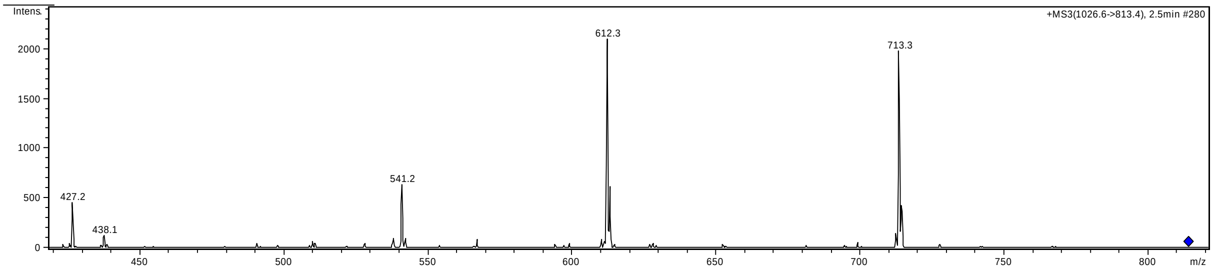

MS^4 (713.3 → x)

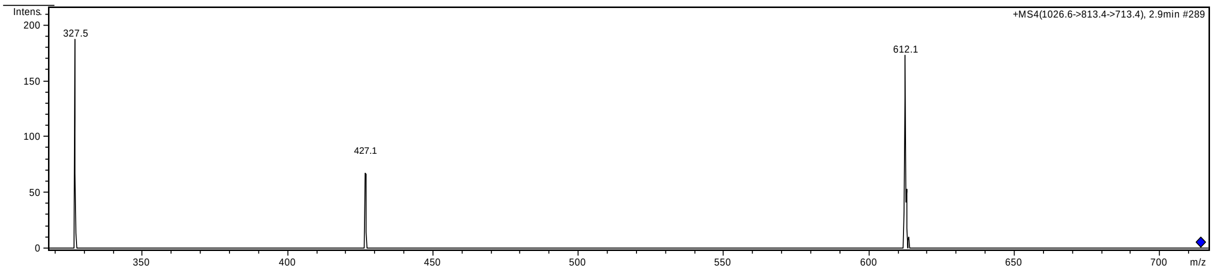

MS^5 (612.1 → x)

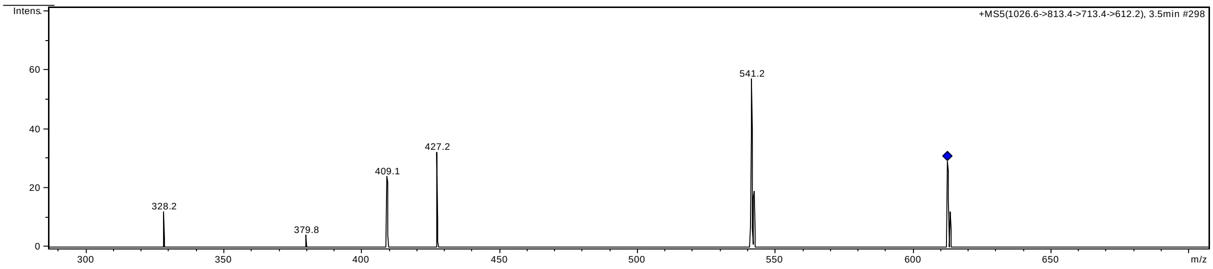

MS^6 (541.2 → x)

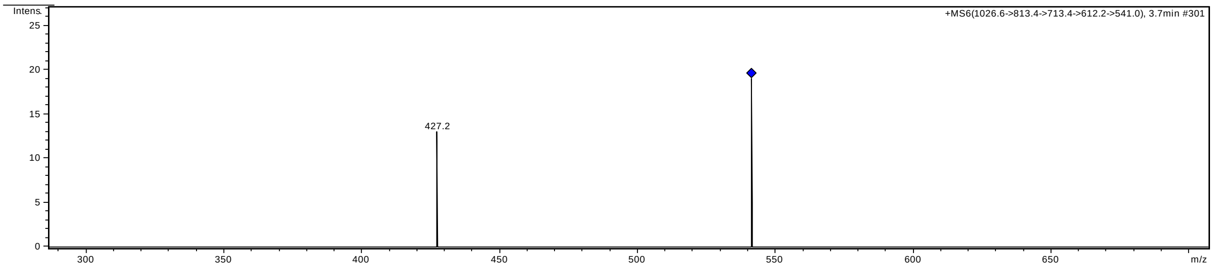

f) Decadepsipeptide (**DP10b**)

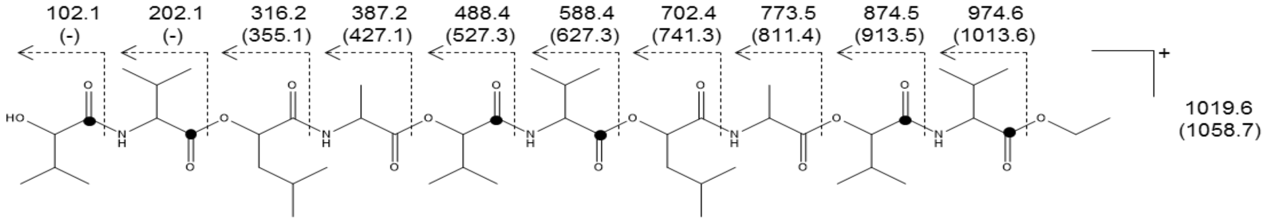

MS

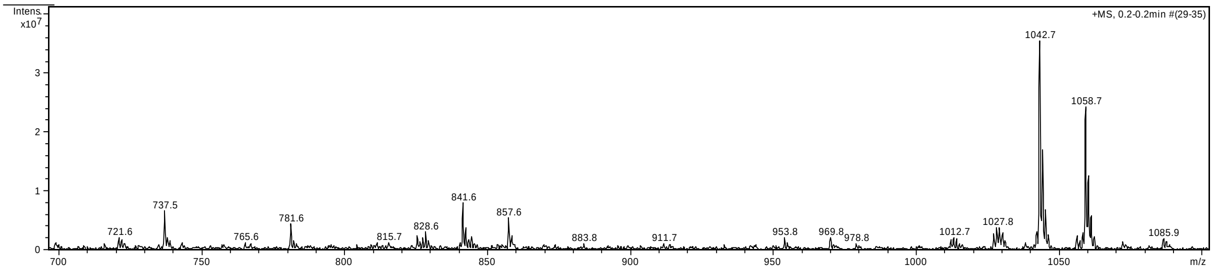

MS^2 (1058.7 → x)

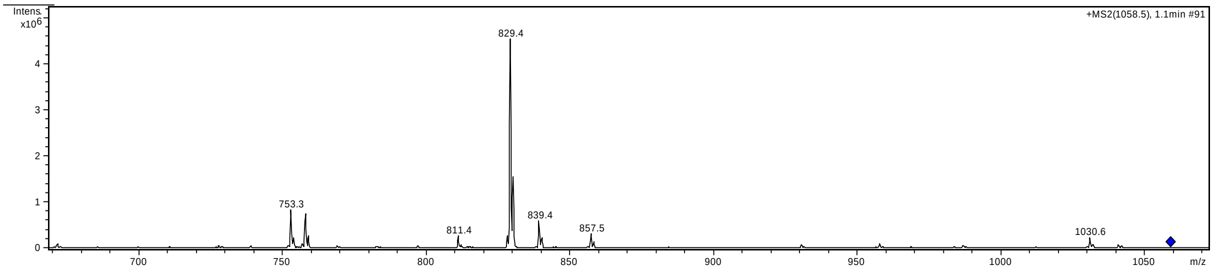

MS^3 (811.4 → x)

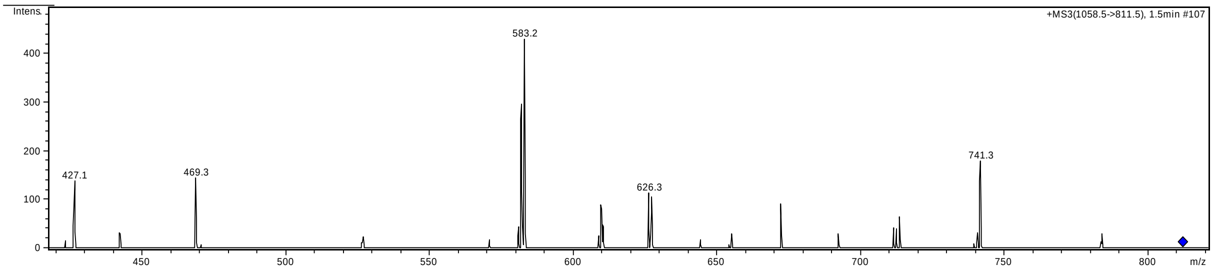

MS^4 (741.3 → x)

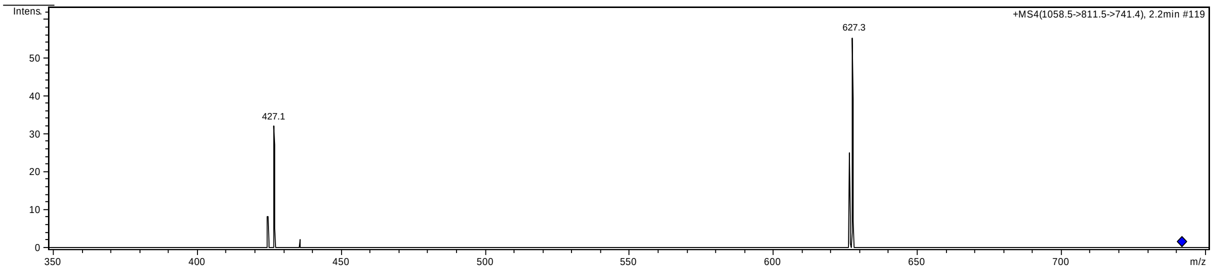

MS^5 (627.3 → x)

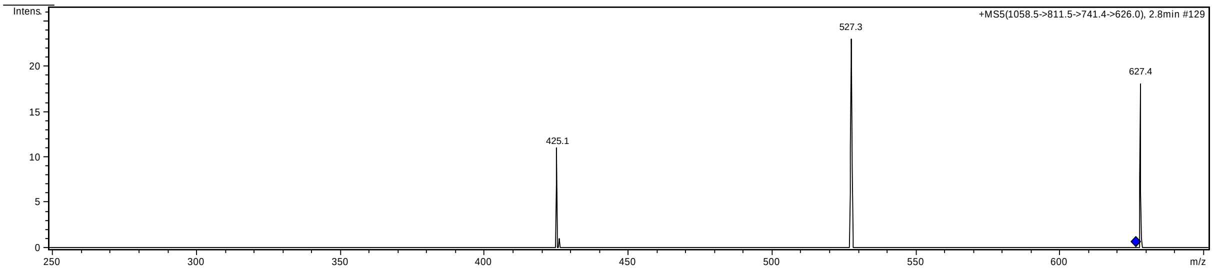

MS^6 (527.3 → x)

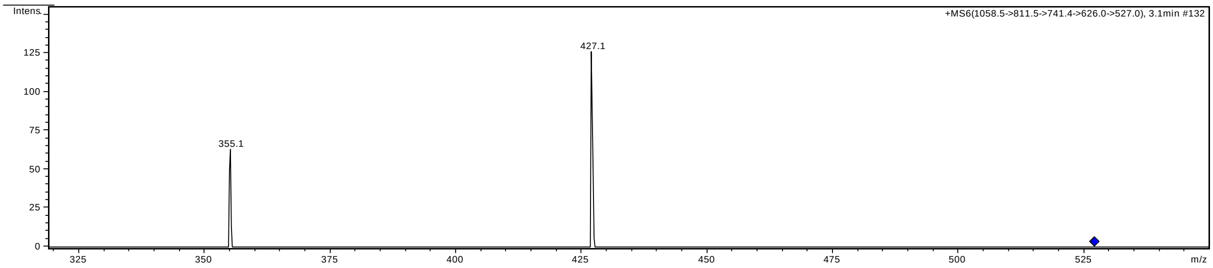

g) Dodecadepsipeptide (**DP12**)

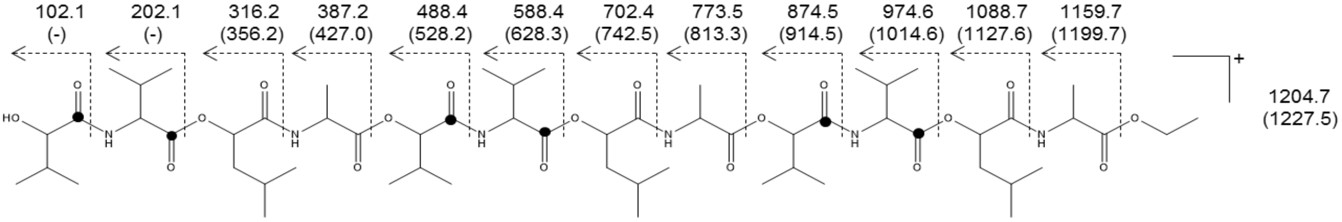

MS

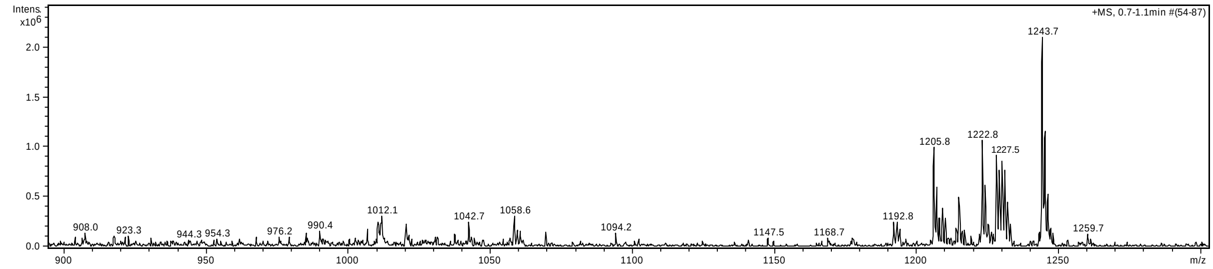

MS^2 (1227.5 → x)

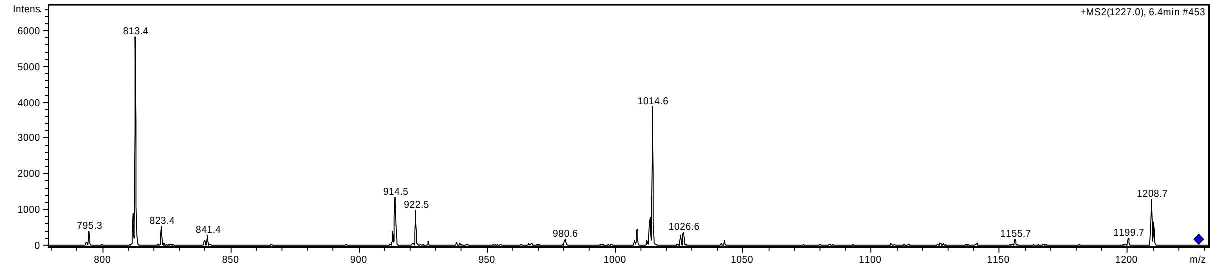

MS^3 (1014.6 → x)

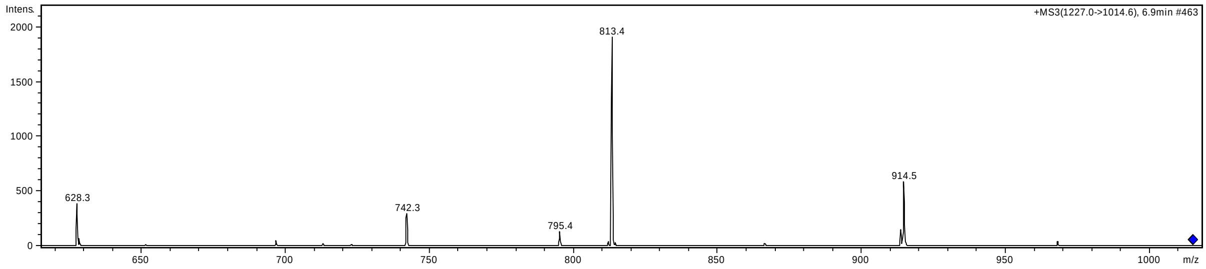

MS^4 (914.5 → x)

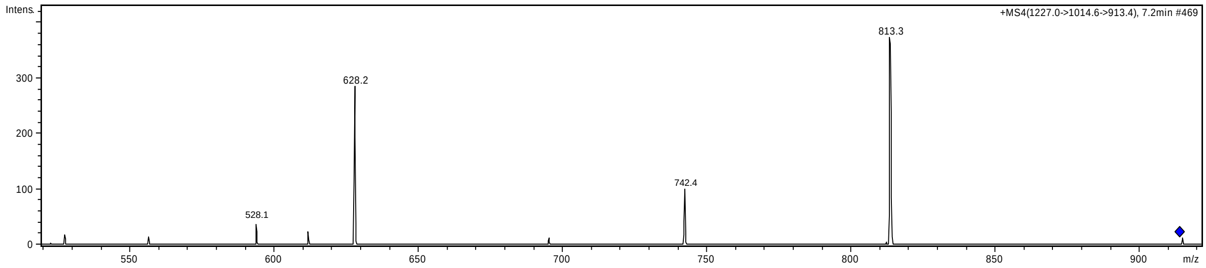

MS^5 (813.3 → x)

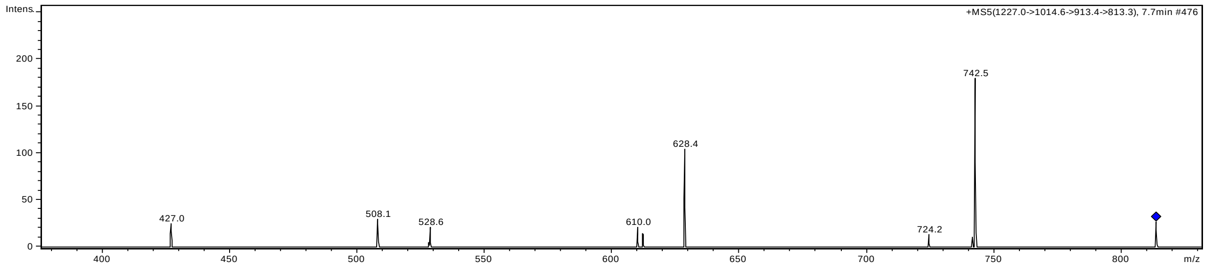

MS^6 (742.5 → x)

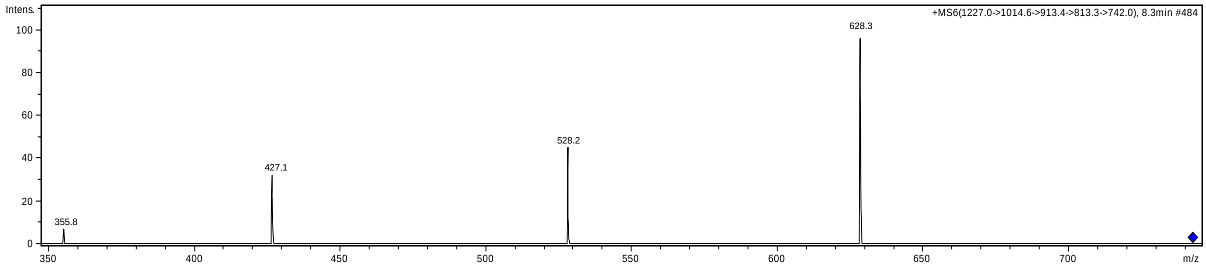

MS^7 (628.3 → x)

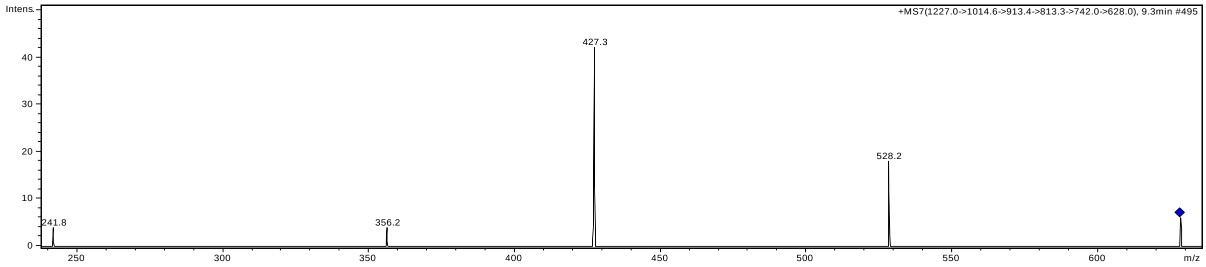

MS^8 (528.2 → x)

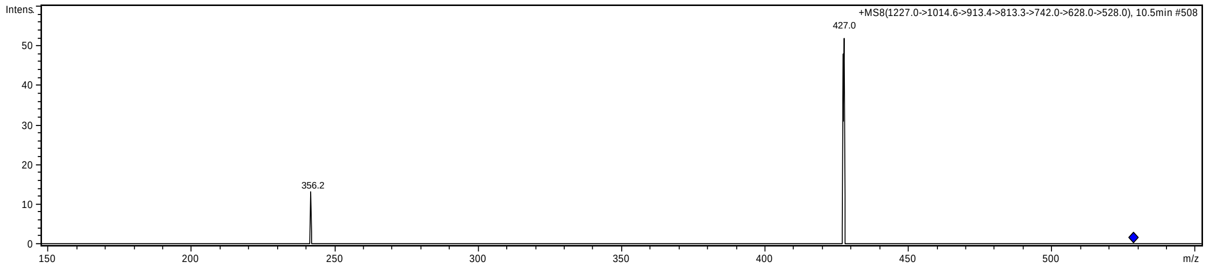

Figure S2 UPLC-TOF MS (ESI^+) analysis of $^{13}C_6$-cereulide and cereulide in a *B. cereus* culture (F4810/72) incubated in the presence of synthetic $^{13}C_6$-cereulide (two biological and three analytical replicates each, ±SD).

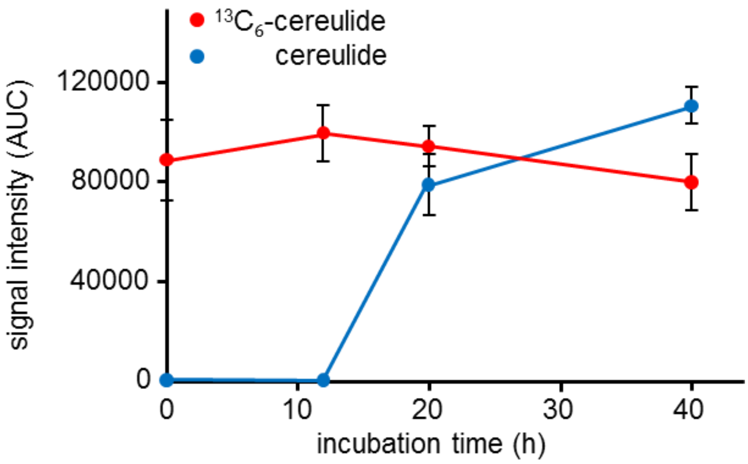

Supplement: Supporting Information [file srep10637-s1.pdf]
